# Thermoneutral housing worsens MASLD and reveals defective brown adipose tissue response to β3-adrenergic stimulation

**DOI:** 10.1016/j.isci.2025.113221

**Published:** 2025-07-26

**Authors:** Céline Marie Pauline Martin, Arnaud Polizzi, Valérie Alquier-Bacquié, Marine Huillet, Clémence Rives, Charlène J.G. Dauriat, Justine Bruse, Valentine Melin, Claire Naylies, Yannick Lippi, Frédéric Lasserre, JingHong Wan, Rémy Flores-Flores, Justine Bertrand-Michel, Florence Blas-Y-Estrada, Elodie Rousseau-Bacquié, Thierry Levade, Hervé Rémignon, Dominique Langin, Etienne Mouisel, Sophie Lotersztajn, Benoit Chassaing, Laurence Gamet-Payrastre, Hervé Guillou, Sandrine Ellero-Simatos, Anne Fougerat, Nicolas Loiseau

**Affiliations:** 1 Toxalim, Université de Toulouse, INRAE, ENVT, EI-Purpan, Toulouse, France; 2 Microbiome-Host Interactions, Institut Pasteur, Université Paris Cité, INSERM U1306, Paris, France; 3 INSERM-UMR1149 Centre de recherche sur l’inflammation, Paris, France; 4 Institut des Maladies Métaboliques et Cardiovasculaires, I2MC, Université de Toulouse, Inserm, Université Toulouse III - Paul Sabatier (UPS), Toulouse, France; 5 Centre Hospitalier Universitaire de Toulouse, Toulouse, France; 6 Institut Universitaire de France (IUF), Paris, France; 7 MetaboHUB-MetaToul, National Infrastructure of Metabolomics and Fluxomics, Toulouse, France

**Keywords:** Endocrine system physiology, Endocrine regulation, Experimental models in systems biology

## Abstract

Metabolic dysfunction-associated steatotic liver disease (MASLD), and its more advanced stage metabolic dysfunction-associated steatohepatitis, is the most common chronic liver disease, constituting a major public health issue. Relevant preclinical models are needed to define molecular mechanisms underlying MASLD pathogenesis and evaluate therapeutic approaches. The majority of the lipids accumulating in the liver upon MASLD originate from adipose tissue and appropriate models to study the liver-adipose tissue dialog are also needed. Here, we demonstrated that, compared to standard temperature housing, thermoneutral housing aggravated western diet (WD)-induced obesity, diabetes, and steatosis in male mice, which was associated with increased hepatic expression of inflammation- and fibrosis-related genes. Accordingly, thermoneutral-housed WD-fed mice developed more severe hepatic inflammation and fibrosis compared to standard-housed mice. We next used thermoneutral-housed WD-fed mice to question the effect of MASLD during β3-adrenergic stimulation. We found that diet-induced MASLD is associated with defective inter-organ metabolic cross-talk which leads to impaired activation of brown adipose tissue.

## Highlights

• Thermoneutral housing promotes WD-induced obesity and MASLD in male mice
• Thermoneutral housing fosters WD-induced changes in hepatic gene expression
• Thermoneutral housing fosters hepatic inflammation and fibrosis
• MASLD is associated with defective BAT response to β3-adrenergic stimulation

## Graphical abstract

**Figure:**
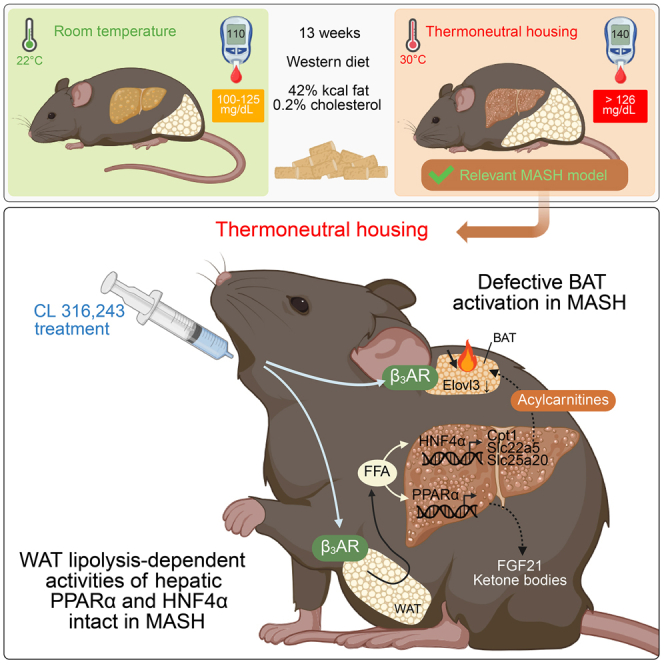

## Introduction

Metabolic dysfunction-associated steatotic liver disease (MASLD) is the most common chronic hepatic liver disease. It comprises several pathologies—ranging from steatosis, which is benign and reversible, to the more severe steatohepatitis, which is characterized by inflammation, hepatocyte damage, and progressive fibrosis, and is a predisposing factor for cirrhosis and hepatocellular carcinoma.^1^^,^^2^ A hallmark of MASLD pathogenesis is hepatic triglyceride accumulation. Free fatty acids (FFAs) release from adipose tissue lipolysis is the primary mechanism contributing to lipid overload within hepatocytes, followed by increased hepatic fatty acid synthesis through *de novo* lipogenesis and, to a lesser extent, dietary fat intake.^3^^,^^4^ MASLD is closely associated with metabolic dysfunctions—including insulin resistance, type 2 diabetes, and obesity—hence the recent name change from non-alcoholic fatty liver disease (NAFLD) to MASLD.^5^^,^^6^^,^^7^ In obese insulin-resistant individuals, insulin cannot suppress adipose tissue lipolysis, and the released FFAs reach the liver. Quantitative lipidomic analysis of both serum and liver has demonstrated that lipids derived from adipocyte lipolysis drive profound changes of lipid remodeling in the mouse liver.^8^ Additionally, adipose tissue secretes cytokines and adipokines that impact liver metabolism and inflammation.^9^

Notably, the liver also impacts the functions of adipose tissue. For example, hepatic peroxisomal β-oxidation has recently been identified as a key regulator of adipocyte browning in diet-induced obesity, through the accumulation of long-chain fatty acids.^10^ The liver plays a critical role in the inter-organ metabolic response to β3-adrenergic stimulation, which leads to triglyceride lipolysis in white adipocytes and thermogenesis activation in brown adipocytes. Activation of β3-adrenergic signaling induces major changes in hepatic gene expression.^11^^,^^12^ Simcox et al. demonstrated that HNF4α activation induces production of liver-derived acylcarnitines that act as alternative fuel for brown adipose tissue (BAT) thermogenesis during cold exposure.^11^ Moreover, β3-adrenergic receptor stimulation induces PPARα-dependent responses in the liver. Hepatocyte PPARα influences insulin secretion and BAT activation induced by β3-adrenergic activation.^12^ The interplay between adipose tissue and the liver plays a crucial role in MASLD initiation and progression.

MASLD is a major public health concern; however, research tools are lacking. Preclinical models are essential for not only investigating the disease mechanisms but also for testing potential therapies and identifying biomarkers. Existing preclinical mouse models of MASLD are generated using dietary challenges or genetic manipulations and only partially recapitulate the human disease, exhibiting minimal or no fibrosis, and/or lacking obesity or even exhibiting weight loss.^13^^,^^14^^,^^15^^,^^16^

Among factors contributing to MASLD development and progression, environmental temperature has emerged as a critical variable in the modeling of human disease. Several studies have highlighted that housing temperature influences mouse physiology.^17^ Most rodent studies are conducted at environmental temperatures that correspond to the thermoneutral zone of dressed adult humans, but that are below thermoneutrality for mice. This results in chronic cold stress, which induces catecholamine release by the sympathetic nervous system, leading to BAT activation and non-shivering thermogenesis to increase heat production and maintain a constant core body temperature. Under these conditions, mice exhibit increase of heart rate, blood pressure, and overall energy expenditure.^18^^,^^19^ Moreover, standard housing suppresses immune responses.^20^^,^^21^ On the other hand, thermoneutral housing accelerates metabolic inflammation in white adipose tissue and in the vasculature during obesity, promoting atherosclerosis progression in mice.^21^^,^^22^ Housing temperature can impact various diseases and treatment responses,^23^ including tumor growth and responses to immune and cytotoxic therapies.^24^^,^^25^

As housing temperature has major influences on metabolism and inflammation—which are both involved in MASLD—thermoneutral housing may improve the modeling of preclinical murine models and translatability to the human disease. Few prior studies have investigated how thermoneutral housing influences MASLD development and progression, and the results are controversial. In one study, male mice were housed at thermoneutrality and fed a high-fat diet for 24 weeks, which resulted in accelerated MASLD with enhanced hepatic steatosis, and increased expression of genes involved in hepatic inflammation and fibrosis, compared to standard temperature-housed mice. That study also found that thermoneutral housing enabled obesity and MASLD experimental modeling in female mice, which are typically resistant to obesogenic diet-induced disease development with standard-temperature housing.^26^ Similarly, another study demonstrated that thermoneutral housing of mice exacerbated liver inflammation induced by a diet rich in fat, carbohydrates, and cholesterol.^27^ Moreover, the combination of thermoneutral housing with a high-fat high-fructose diet for mice has been shown to recapitulate many of the histological and genomic characteristics of advanced human MASLD.^28^ In contrast, another study reported that thermoneutral housing coupled with western diet (WD) feeding did not aggravate diet-induced hepatic inflammation and fibrosis in male or female mice.^29^ Horakova et al. suggested that the effect of housing temperature may depend on mouse genetic background.^30^ Overall, the effect of thermoneutrality on diet-induced MASLD remains unclear due to the use of different experimental conditions, including mouse strains, and diet composition and duration.

In the present study, we investigated the impact of thermoneutral housing on a WD-induced mouse model of MASLD.^15^^,^^16^ We characterized the effects of environmental temperature on whole-body homeostasis, liver phenotype, and hepatic gene expression. Furthermore, we used MASLD model mice housed at thermoneutrality to investigate the metabolic cross-talk between adipose tissues and the liver during lipolysis induced by β3-adrenergic stimulation.

## Results

### Thermoneutral housing promotes WD-induced obesity, diabetes, and steatosis

**Figure 1:**
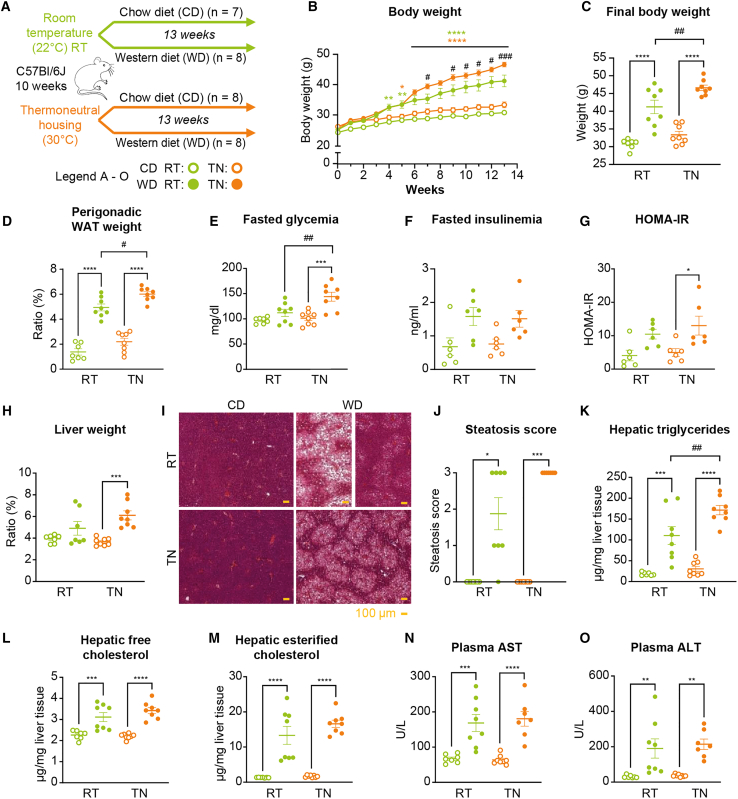
Thermoneutral housing promotes WD-induced obesity, diabetes, and steatosis (A) WT male C57Bl/6J mice, aged 10 weeks, were housed at room temperature (RT; 22°C) or thermoneutral temperature (TN; 30°C) and fed a chow diet (CD) or western diet (WD) for 13 weeks (*n* = 7–8/group). (B) Body weight was determined weekly during the experiment. (C) Final weight after 13 weeks of diet. (D) Ratio of perigonadal (PG) white adipose tissue (WAT) weight to body weight at the end of the experiment. (E and F) Fasted glycemia (E) and insulinemia (F) measured after 6 h of fasting. (G) Homeostatic model assessment of insulin resistance (HOMA-IR). (H) Ratio of liver weight to body weight at the end of the experiment. (I) Representative histological sections of liver, stained with H&E, from each group at 10×. WD-RT mice displayed high heterogeneity in steatosis development, thus 2 sections were chosen as representative for this group. Scale bars, 100 μm. (J) Liver steatosis estimated on histological liver sections. Scoring: parenchymal involvement by steatosis <5%, 0; 5%–33%, 1; 33%–66%, 2; >66%, 3 (*n* = 7–8/group) (K–M) Hepatic neutral lipids (triglycerides, free cholesterol, and esterified cholesterol) extracted from livers, and analyzed by gas-liquid chromatography. (N and O) Plasma alanine aminotransferase (ALT) and aspartate aminotransferase (AST) activity. Data are presented as the mean ± SEM for *n* = 7–8/group. ∗diet effect; #temperature effect between WD groups; ∗ or #*p* < 0.05; ∗∗ or ##*p* < 0.01; ∗∗∗ or ###*p* < 0.001; ∗∗∗∗ or ####*p* < 0.0001. Differential effects were analyzed by analysis of variance (one or two-way ANOVA) with post-hoc Šídák’s test. Histological scores (K) were analyzed using a non-parametric test (Kruskall-Wallis).

To investigate the effect of environmental temperature on diet-induced MASLD, male mice were housed at 22°C (standard temperature, RT) or 30°C (thermoneutral housing, TN), and fed a chow (CD) or WD for 13 weeks (Figures 1A and S1). After 5 weeks, WD-fed mice were significantly heavier than CD-fed mice. After 9 weeks, TN-housed WD-fed mice were significantly heavier than RT-housed WD-fed mice (Figure 1B). Housing temperature significantly impacted WD-induced obesity, as confirmed by final total body weight and relative white adipose tissue (WAT) weight (Figures 1C and 1D). Whereas no difference in fasted glycemia was observed between RT-housed CD-fed vs. WD-fed mice, TN-housed WD-fed mice displayed significantly higher fasting glycemia compared to both TN-housed CD-fed mice and RT-housed WD-fed mice (Figure 1E). Fasted insulinemia did not significantly differ between groups (Figure 1F). This resulted in a significantly increased homeostatic model assessment of insulin resistance (HOMA-IR) among TN-housed WD-fed mice only, compared to their CD-fed controls (Figure 1G).

We next investigated the hepatic phenotype. Liver weight was significantly higher in TN-housed WD-fed animals compared to CD-fed mice but did not differ between the groups housed at RT (Figure 1H). Histological H&E staining revealed that WD feeding induced hepatic steatosis, regardless of housing temperature. However, responses to the WD showed more heterogeneity at RT (Figure 1I). The steatosis scores confirmed significant lipid accumulation upon WD at both RT and TN, and the more heterogeneous response to WD at RT compared to at TN (Figure 1J). These findings were further supported by the quantification of hepatic triglycerides (Figure 1K). Additionally, both hepatic free cholesterol and esterified cholesterol were higher in the WD group compared to the CD group, with no significant influence of housing temperature (Figures 1L and 1M). Similarly, significantly greater liver damage was observed in WD-fed compared to CD-fed mice, regardless of housing temperature (Figures 1N and 1O).

Overall, these results suggested that TN housing potentiated WD-induced obesity, glucose intolerance, and hepatic steatosis development. However, the effect of TN on these parameters was much less pronounced than that of WD.

### Thermoneutral housing fosters WD-induced changes in hepatic gene expression

**Figure 2:**
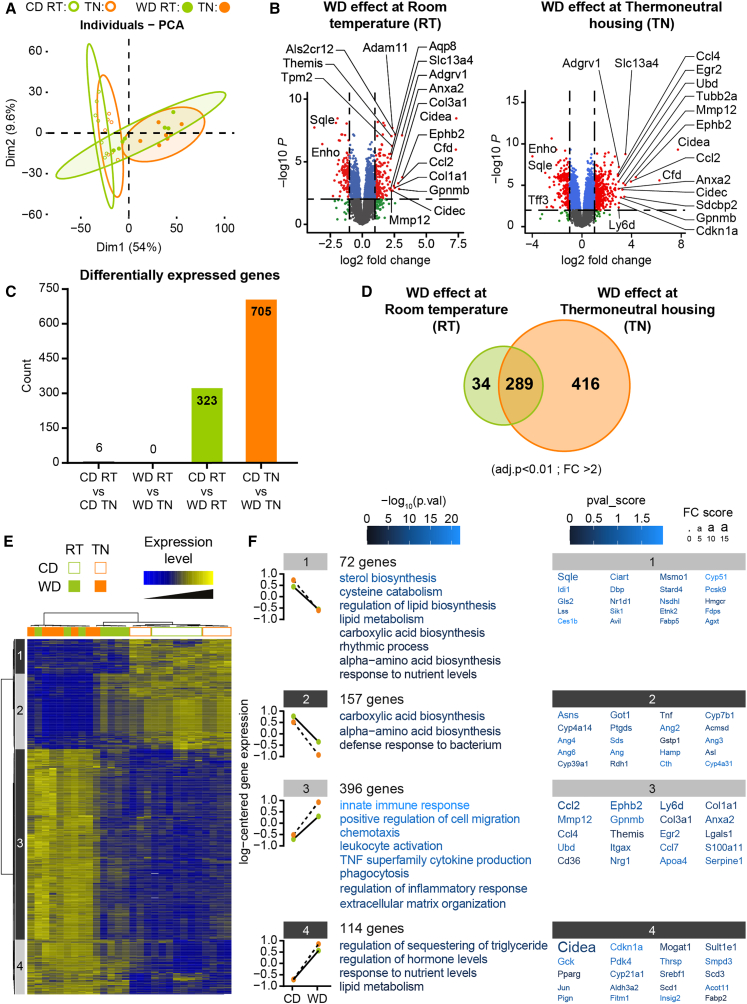
Thermoneutral housing fosters WD-induced changes in hepatic gene expression (A) Principal-component analysis (PCA) score plots of whole-liver transcriptome datasets (*n* = 7–8/group). Each dot represents an observation (animal), projected onto the first (horizontal axis) and second (vertical axis) PCA variables. (B) Volcano plot shows effects of western diet (WD) on gene expression under room temperature (RT; left panel) or thermoneutral temperature (TN; right panel). Each gene expression level is shown in terms of the –log_10_*p* value, for comparisons between the WD group and the chow diet group at each temperature. The –log_10_*p* values are plotted as a function of the associated log_2_-fold change, or formally, log_2_(WD)-log_2_(Chow). Red indicates *p* < 0.01 and log_2_(fold change) > 2. Blue indicates *p* < 0.01 and log_2_ (fold change) < 2. Green indicates *p* > 0.01 and log_2_(fold change) > 2. Gray indicates *p* > 0.01 and log_2_(fold change) < 2. Gene names are highlighted for the 20 most highly regulated genes, according to a score based on the adjusted *p* value × logFC. (C) Histogram represents the number of genes significantly regulated for each comparison. (D) Venn diagram represents the number of genes significantly regulated by WD (*p* < 0.001, FC > 2) for each housing temperature (RT or TN). (E) Heatmap represents data from microarray experiments. Significantly differentially expressed genes (adjusted *p* values <0.01 and fold change >2) were selected, which corresponded to 739 probes. The color gradient indicates the scaled values of gene expression. Hierarchical clustering identified four gene clusters (indicated on the left). (F) Mean expression profiles for the four gene clusters. Graphs represent the means of the scaled gene expression values. The most significantly enriched biological processes, identified using the Metascape gene ontology algorithm, are shown at the right of each profile. Briefly, hypergeometric tests were performed for each category in each cluster. The top 20 genes in each cluster that showed the largest differences in expression. The color of each character string is related to the *p* value score. The size of each character string is related to the fold change score for all the comparisons made for each gene.

We next performed microarray analysis of liver gene expression to identify biological processes sensitive to housing temperature under CD or WD feeding (Table S1). Principal-component analysis (PCA) of the whole-liver transcriptome revealed clear separation between the CD-fed and WD-fed groups along the first principal component, accounting for 54% of the variance (Figure 2A). Interestingly, compared to the TN-housed WD-fed animals, the RT-housed WD-fed animals exhibited less clear clustering, with four RT-housed WD-fed individuals clustering close to the CD-fed animals. Our results showed overlap between CD-fed mice at RT and TN, and the same phenomenon was observed among WD-fed mice. Volcano plots of the effect of WD showed that the genomic response to WD was stronger at TN than RT (Figure 2B). Indeed, 705 hepatic genes exhibited significantly modulated expression between WD-fed and CD-fed animals at TN, while only 323 genes were differentially expressed according to WD at RT (Figure 2C). The Venn diagram of these differentially expressed genes (DEGs) showed that among the genes affected by WD at RT, the vast majority were also affected by WD at TN (Figure 2D). Hierarchical clustering using the 739 DEGs confirmed the marked discrimination between WD-fed and CD-fed mice (Figure 2E). Also, in accordance with the PCA results, four RT-housed WD-fed individuals were closer to the CD-fed mice than to the other WD-fed mice.

Next, gene clustering revealed four gene clusters along the vertical axis of the heatmap. In the first cluster, 72 genes exhibited lower mRNA expression among WD-fed compared to CD-fed mice, regardless of housing temperature. This cluster was most significantly associated with the biological processes of “sterol biosynthesis” (*p* = 10^−15^) and “regulation of lipid biosynthesis” (*p* = 10^−7^). The most significantly affected genes in cluster 1 included genes involved in cholesterol biosynthesis, such as *Hmgcr*, *Sqle*, *Lss*, and *Msmo1*. The 114 genes in cluster 4 were also upregulated by the WD, regardless of housing temperature. Cluster 4 genes were associated with “sequestering of triglycerides” (*p* = 10^−6^) and “lipid metabolism” (*p* = 10^−6^), and included *Cidea* and *Fitm1*, which are involved in triglyceride storage by promoting lipid droplet formation, and *Thrsp*, which regulates the triglyceride biosynthetic process.

On the other hand, the 157 genes in cluster 2 exhibited lower mRNA expression among WD-fed compared to CD-fed mice, which was greater at TN than at RT. Interestingly, among the four RT-housed WD-fed mice that clustered close to the CD-fed animals, the mRNA expression levels of cluster 2 genes were similar to those in the CD-fed mice. Cluster 2 genes were most significantly associated with the biological process of “carboxylic acid biosynthesis” (*p* = 10^−8^). Finally, cluster 3 included 396 genes that exhibited increased expression in WD-fed compared to CD-fed mice, and this difference was temperature-dependent. Notably, as seen for cluster 2, the WD-induced increase in mRNA expression was not observed in the four RT-housed WD-fed mice that clustered with the CD-fed animals. Interestingly, the cluster 3 genes were involved in inflammation and fibrosis with the most significantly affected pathways included “innate immune response” (*p* = 10^−22^), “regulation of inflammatory response” (*p* = 10^−12^), and “extracellular matrix organization” (*p* = 10^−13^). Genes contributing to these pathways included genes encoding for pro-inflammatory cytokines (*Ccl2*, *Ccl4*, and *Ccl7*), for collagens (*Col1a1* and *Col3a1*), and for extra-cellular matrix-degrading metalloproteinases (*Mmp12*) (Figure 2F).

Taken together, these analyses illustrated that WD feeding induced more changes in hepatic gene expression at TN compared to at RT, particularly among genes involved in pro-inflammatory and pro-fibrotic pathways.

### Thermoneutral housing fosters WD-induced hepatic inflammation and fibrosis

**Figure 3:**
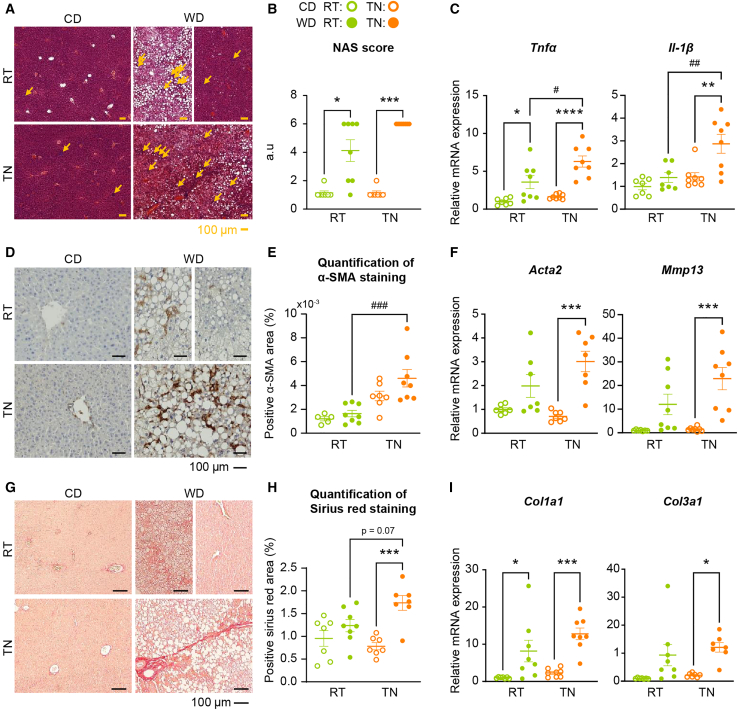
Thermoneutral housing fosters WD-induced hepatic inflammation and fibrosis (A) Representative histological sections of liver stained with H&E from each group at 10×. WD-RT mice displayed high heterogeneity in steatosis development, thus 2 sections were chosen as representative for this group. Scale bars, 100 μm. (B) NAFLD activity score (NAS). (C) Hepatic mRNA expression of inflammatory genes (*Tnfα* and *Il-1β*) measured by RT-qPCR. (D) Representative histological sections of liver from each group, evaluated by immunohistochemical staining with alpha smooth muscle (α-SMA), at 20×. WD-RT mice displayed high heterogeneity in steatosis development, thus 2 sections were chosen as representative for this group. Scale bars, 100 μm. (E) Stellate hepatic cell activation estimated on histological liver sections by quantification of α-SMA staining (*n* = 7–8/group). (F) Hepatic mRNA expression of genes involved in stellate cell activation (*Acta2* and *Mmp13)* measured by RT-qPCR. (G) Representative histological sections of liver from each group, stained with Sirius red, at 10×. WD-RT mice displayed high heterogeneity in steatosis development, thus 2 sections were chosen as representative for this group. Scale bars, 100 μm. (H) Liver fibrosis estimated on histological liver sections by quantification of Sirius Red (collagen) staining (*n* = 7–8/group). (I) Hepatic mRNA expression of fibrosis genes (*Col1a1* and *Col3a1*) measured by RT-qPCR. Data are presented as the mean ± SEM for *n* = 7–8/group. ∗diet effect; #temperature effect; ∗ or #*p* < 0.05; ∗∗*p* < 0.01; ∗∗∗ or ###*p* < 0.001; ∗∗∗∗*p* < 0.0001. Differential effects were analyzed by analysis of variance (one-way ANOVA) with post-hoc Šídák’s test (B, E, and H) Histological scores were analyzed using a non-parametric test (Kruskall-Wallis).

Histological analysis was performed to quantify liver inflammation and fibrosis. Blinded assessment of H&E-stained histological sections and calculation of the NAFLD activity score (NAS), which includes liver steatosis and lobular inflammation, revealed greater infiltration of inflammatory cells in the liver of WD-fed mice, compared to CD-fed animals (Figures 3A and 3B). Again, mice at RT exhibited a heterogeneous response to the WD, which was not observed at TN. These findings correlated with the increased hepatic expressions of *Tnfα* and *Il1β* (Figure 3C). We next assessed fibrosis by alpha smooth muscle cell actin (α-SMA) staining, which indicated stellate cell activation, as well as Sirius red staining, which marked type I and II collagen fibers (Figures 3D and 3G). α-SMA staining was significantly more abundant in TN-housed WD-fed mice, compared to RT-housed WD-fed mice (Figure 3E). Collagen was significantly more abundant in TN-housed WD-fed mice, compared to TN-housed CD-fed mice, while we observed no significant difference between WD-fed and CD-fed mice at RT (Figure 3H). These findings correlated with the increased gene expressions of *Acta2*, *Mmp13*, *Col1a1*, and *Col3a1* in TN-housed WD-fed mice (Figures 3F and 3I).

Altogether, these results revealed that TN housing aggravated WD-induced inflammation and fibrosis, suggesting an acceleration of MASLD progression.

### Thermoneutral housing reduces the intra-group variability in WD-induced MASLD

Upon finding a more heterogeneous response to WD feeding under RT compared to TN conditions, we wondered whether the heterogeneous response observed at RT might be due to a cage effect. However, each cage of RT-housed WD-fed mice included two individuals that were “bad responders” and two that were “good responders”, in terms of body weight, liver damage, hepatic steatosis, and fibrosis (Figure S2). To further investigate the TN housing-induced decrease in heterogeneity of response to WD, we first analyzed the hepatic gene variance distribution in our microarray data. Among CD-fed mice, the distribution of gene variances overlapped between RT- and TN-housed mice. On the other hand, among WD-fed mice, the RT-housed mice exhibited a shift of the variance distribution to the right side of the plot, indicating higher intra-group variance in this group compared to among TN-housed mice (Figure S3A). This was confirmed by a decreased mean intra-group variance for the non-homoscedastic genes (i.e., genes showing a significant difference in the intra-group variance between the experimental groups) in TN-housed WD-fed mice compared to RT-housed WD-fed mice, while no significant difference was observed between the groups of CD-fed mice (Figure S3B). Interestingly, most of the genes showing significantly higher intra-group variability in RT-housed WD-fed animals were related to inflammation and fibrosis (Figure S4). Finally, we confirmed that in terms of body weight, liver damage, and hepatic steatosis, the intra-group standard error of mean (SEM) was significantly lower among TN-housed WD-fed mice compared to RT-housed WD-fed mice (Table S2). Overall, this analysis demonstrated that compared to RT housing, TN housing significantly reduced the intra-group variability in response to WD feeding.

### Thermoneutral housing WD-induced MASLD models human MASH

We next compared liver gene expression profiles from our two preclinical MASLD models (RT vs. TN) with human MASLD. We used datasets from 2 previously published human MASLD cohorts^15^^,^^16^: the University of Cambridge (UCAM) and Virginia Commonwealth University (VCU) super-cohort and the Newcastle University (EPoS) cohort, comprising 136 and 168 patients, respectively. Vacca et al. identified DEGs that are prevalent in the early stages of the disease (“mild vs. control”), during MASLD progression (“moderate vs. mild”) or at later MASH stages (“severe vs. mild”). We correlated these DEGs with gene expression changes in the liver of WD-fed vs..CD-fed mice at RT or at TN (Figure S5). Correlations between WD-induced changes in mouse gene expression and human DEGs were stronger at later disease stages than at early disease stages, for both RT and TN-housing. Moreover, correlations were stronger and more significant between WD-induced change in mouse gene expression and human DEGS at TN than at RT, at all stages of the human disease. Thus, upon WD feeding, TN-housing recapitulated the hepatic gene expression changes observed during human MASLD progression more closely than RT-housing.

### WD-induced MASLD is associated with altered hepatic gene expression in response to β3-adrenergic stimulation

**Figure 4:**
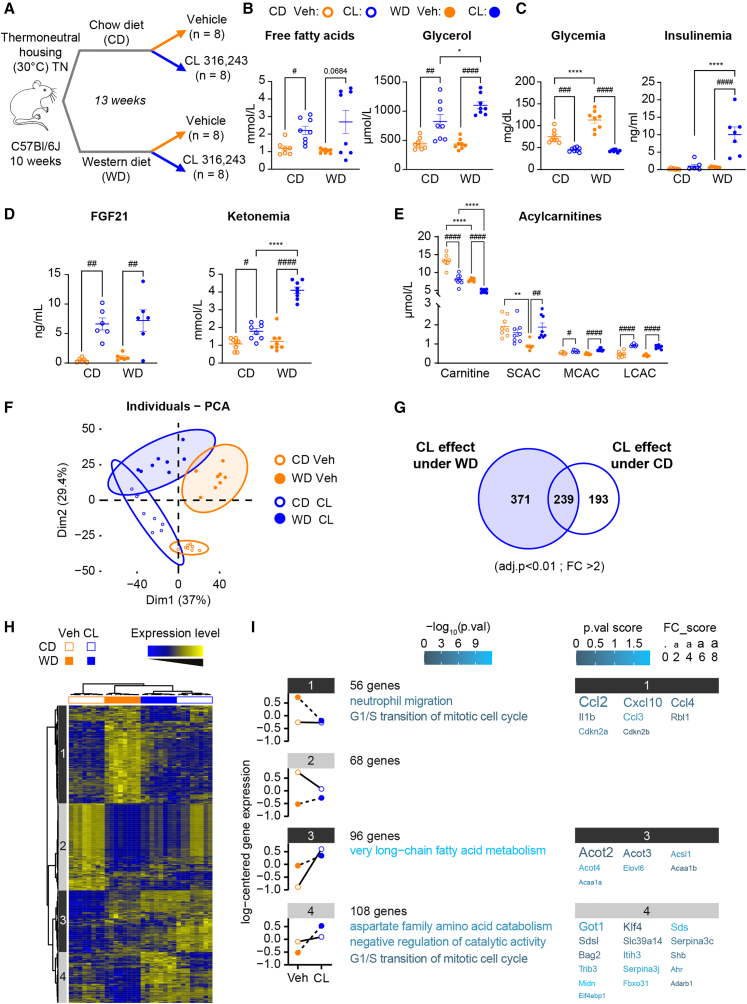
WD-induced MASLD is associated with altered hepatic gene expression in response to β3-adrenergic stimulation (A) WT male C57Bl/6J mice, aged 10 weeks, housed at thermoneutral temperature (TN; 30°C) for 13 weeks and fed a chow diet (CD) or western diet (WD). At the end of experiment, mice fasted at ZT0, were given CL 316,243 or vehicle by gavage at ZT10, and were sacrificed at ZT16. (B) Plasma free fatty acid and glycerol levels. (C) Blood glucose and plasma insulin levels at sacrifice. (D) Circulating levels of Fgf21 and ketone bodies (β-hydroxybutyrate). (E) Plasma levels of carnitine, short-chain acylcarnitines (SCACs) (C2-C5), medium-chain acylcarnitines (MCACs) (C6-C12), and long-chain acylcarnitines (LCACs) (C14-C18) as measured by LC-MS in mice treated with vehicle or CL 316,243. *n* = 8/group. (F) Principal component analysis (PCA) score plots of the whole-liver transcriptome datasets (*n* = 8/group). Each dot represents an observation (animal), projected onto the first (horizontal axis) and second (vertical axis) PCA variables. (G) Venn diagram represents the number of genes significantly regulated by CL for each diet (CD or WD). (H) Heatmap represents data from microarray experiments. Selected genes were significantly differentially expressed, with interaction between the CL effect and the diet (adjusted *p* values <0.05 and fold change >1.5), which corresponded to 332 probes. The color gradient indicates the scaled values of gene expression. Hierarchical clustering identified four gene clusters (indicated on the left). (I) Mean expression profiles for the four gene clusters. Graphs represent the means of the scaled gene expression values. The most significantly enriched biological processes, identified using the Metascape gene ontology algorithm, are shown at the right of each profile. Briefly, hypergeometric tests were performed for each category in each cluster. The top 20 genes in each cluster that showed the largest differences in expression. The color of each character string is related to the *p* value score. The size of each character string is related to the fold change score for all the comparisons made for each gene. Data are presented as the mean ± SEM for *n* = 8/group. ∗diet effect; #CL 316,243 effect; ∗ or #*p* < 0.05; ∗∗*p* < 0.01; ∗∗∗ or ###*p* < 0.001; ∗∗∗∗ or ####*p* < 0.0001. Differential effects were analyzed by analysis of variance (one-way ANOVA) with post-hoc Šídák’s test.

During MASLD development, FFAs released by WAT lipolysis are the major source of hepatic fatty acid accumulation.^3^^,^^4^ Moreover, acute activation of adipocyte lipolysis has been shown to induce major changes in hepatic gene expression.^11^^,^^12^ We recently demonstrated that adipocyte ATGL-dependent lipolysis controls liver gene expression in response to fasting or β3-adrenergic stimulation, notably PPARα-dependent gene expression and hepatic ketone body and FGF21 production.^12^ Cold-induced lipolysis also induces HNF4α-dependent expression of enzymes involved in acylcarnitine metabolism in the liver.^11^ However, whether these lipolysis-induced hepatic gene expression changes are still present in the context of MASLD has not been investigated. Thus, we took advantage of our nutritional MASLD model to investigate this dialog between the WAT and the liver. We induced adipocyte lipolysis through β3-adrenergic receptor activation using CL 316,243 (CL) in fasted male mice housed at TN and fed the CD or WD (Figures 4A and S1). After CL treatment, both CD-fed and WD-fed mice exhibited significantly increased FFA and glycerol levels (Figure 4B). Both diet groups showed decreased blood glucose levels following CL treatment, but the insulin levels after CL-induced β3-adrenergic signaling activation were higher in WD-fed mice than in CD-fed mice (Figure 4C). We measured the hepatic expressions of PPARα- and HNF4α-target genes, which were increased in response to CL among both CD- and WD-fed mice (Figure S6). Accordingly, plasma levels of ketone bodies, FGF21, and acylcarnitines were induced in CL-treated mice regardless of the diet (Figures 4D and 4E).

We next assessed liver gene expression using microarray analysis, to identify biological processes that were sensitive to CL treatment under CD or WD feeding (Table S3). PCA of the whole hepatic transcriptome revealed clear separation between the vehicle and CL groups along the first principal component, accounting for 37% of the variance. We also observed discrimination between the CD-fed and WD-fed groups along the second principal component, accounting for 29.4% of the variance (Figure 4F). A Venn diagram showed that CL treatment affected a set of genes shared among CD-fed and WD-fed mice, but also diet-specific genes, especially in response to WD (Figure 4G). Therefore, we performed hierarchical clustering using the 332 genes that displayed a significant interaction between CL treatment and diet (Figure 4H). The gene clustering revealed four major genetic groups along the vertical axis of the heatmap (Figure 4I). In cluster 1, 56 genes displayed lower expression in CL-treated vs. vehicle-treated mice, only among WD-fed mice. These genes were notably involved in the biological process of “neutrophil migration” (*p* = 10^−5^), and included genes for chemokines, such as *Ccl2*, *Cxcl10*, and *Ccl4*. Similarly, cluster 4 included 104 genes that showed higher expression in CL-treated than in vehicle-treated mice, only among WD-fed mice. This cluster showed involvement in “aspartate family amino acid catabolism” (*p* = 10^−5^). In contrast, the 68 genes from cluster 2, and the 96 genes from cluster 3, were significantly impacted upon CL treatment in CD-fed mice but not in WD-fed mice. Interestingly, cluster 3 genes that exhibited higher expression in CL-treated vs. vehicle-treated CD-fed mice were involved in “very long-chain fatty acid metabolism” (*p* = 10^−10^).

Collectively, these results demonstrated that WD-induced MASLD is associated with altered hepatic gene expression in response to β3-adrenergic stimulation that was not dependent on hepatic PPARα and HNF4α activities or due to defective adipose lipolysis.

### WD-induced MASLD is associated with defective BAT activation in response to β3-adrenergic stimulation

**Figure 5:**
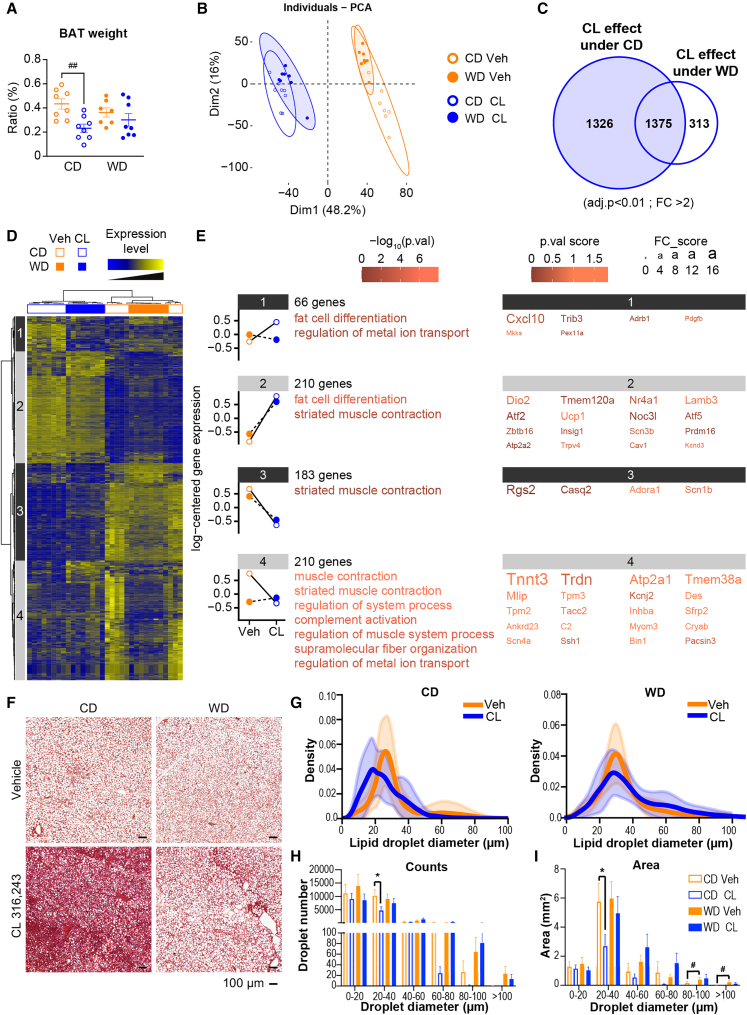
WD-induced MASLD is associated with defective BAT activation in response to β3-adrenergic stimulation (A) Ratio of brown adipose tissue (BAT) to body weight at the end of the experiment. (B) Principal-component analysis (PCA) score plots of the whole-BAT transcriptome datasets (*n* = 8/group). Each dot represents an observation (animal), projected onto the first (horizontal axis) and second (vertical axis) PCA variables. (C) Venn diagram represents the number of genes significantly regulated by CL for the chow diet (CD) or western diet (WD). (D) Heatmap represents data from microarray experiments. The selected genes were significantly differentially expressed, with interaction between the CL effect and the diet (adjusted *p* values <0.05 and fold change >1.5), which corresponded to 669 probes. The color gradient indicates the scaled values of gene expression. Hierarchical clustering identified four gene clusters (indicated on the left). (E) Mean expression profiles for the four gene clusters. Graphs represent the means of the scaled gene expression values. The most significantly enriched biological processes, identified using the Metascape gene ontology algorithm, are shown at the right of each profile. Briefly, hypergeometric tests were performed for each category in each cluster. The top 20 genes in each cluster that showed the largest differences in expression. The color of each character string is related to the *p* value score. The size of each character string is related to the fold change score for all the comparisons made for each gene. (F) Representative histological sections of BAT stained with H&E from each group at 10×. Scale bars 100 μm. (G) Lipid droplet distribution according to their size. (H) Lipid droplet counts. (I) Lipid droplet area. Data are presented as the mean ± SEM for *n* = 8/group. ∗diet effect; #CL 316,243 effect; ∗ or #*p* < 0.05; ∗∗*p* < 0.01; ∗∗∗ or ###*p* < 0.001; ∗∗∗∗ or ####*p* < 0.0001. Differential effects were analyzed by analysis of variance (one-way ANOVA) with post-hoc Šídák’s test. (F) Histological scores were analyzed using a non-parametric test (Kruskall-Wallis).

β3-adrenergic signaling induces triacylglycerol lipolysis in WAT, as well as activates thermogenesis in BAT.^31^^,^^32^^,^^33^^,^^34^ Again, we took advantage of our TN-housing WD-induced MASLD preclinical model to investigate the consequences of MASLD on BAT activation, in response to high-level lipolysis induced by combined CL treatment and fasting (Figure S1). Compared to their vehicle counterparts, CL-treated CD-fed mice exhibited significantly lower BAT weight, while CL-treatment did not significantly impact BAT weight among WD-fed mice (Figure 5A). Microarray analysis of BAT gene expression revealed biological processes that were sensitive to CL treatment under CD or WD feeding (Table S4). PCA of the whole BAT transcriptome showed clear separation between vehicle- and CL-treated mice along the first principal component, accounting for 48.2% of the variance. On the other hand, no significant discrimination was observed between CD-fed and WD-fed mice on any principal component (Figure 5B). However, a Venn diagram showed that CL treatment affected a much higher number of genes in CD-fed mice (2,701 DEGs) than in WD-fed mice (1,688 DEGs) (Figure 5C). We confirmed that *Elovl3* mRNA expression was significantly decreased in CL-treated WD-fed mice compared to CL-treated CD-fed mice, and that other BAT markers exhibited a lower fold change of induction upon CL treatment among WD-fed mice compared to CD-fed mice^35^ (Figure S7).

We performed hierarchical clustering using the 669 genes showing a statistically significant interaction between CL treatment and diet, which confirmed the marked discrimination between CL-treated and vehicle-treated mice, regardless of diet, as previously observed by PCA (Figure 5D). Subsequent gene clustering revealed four major genetic groups along the vertical axis of the heatmap. Interestingly, in all clusters, the effect of CL treatment observed in CD-fed mice was significantly lower (clusters 2 and 3) or completely blunted (clusters 1 and 4) in WD-fed mice. Among the genes that exhibited a decreased response to CL-induced lipolysis in WD-fed mice, some were involved in “fat cell differentiation” (clusters 1 and 2), including genes related to brown adipocyte differentiation and thermogenesis, such as *Dio2*, *Ucp1*, and *Prdm16* (Figure 5E). Histological staining revealed lower numbers of lipid droplets in CL-treated CD-fed mice compared to in vehicle-treated CD-fed mice; however, this CL-dependent effect was not observed in WD-fed mice (Figure 5F). This finding was further confirmed by measurement of lipid droplet diameter, which revealed a shift toward smaller lipid droplets in the BAT of CL-treated CD-fed mice, compared to vehicle-treated CD-fed mice. In contrast, these two distributions overlapped among WD-fed mice (Figure 5G). Comparisons of the number of lipid droplets aggregated according to size, and of the area occupied by the droplets, further illustrated that CL-treatment significantly decreased the number lipid droplets and the surface occupied by small lipid droplets (20–40 μm) among CD-fed mice but not WD-fed mice (Figures 5H and 5I).

Overall, BAT activation in response to CL-induced β3-adrenergic stimulation was blunted in this mouse model of obesity and MASLD. Previous study reported that in response to acute adipocyte lipolysis, the transcription factors PPARα and HNF4α can influence BAT activation.^11^^,^^12^ However, as shown in Figure 4 and S6, PPARα- and HNF4α-dependent responses were quite similar in CD and WD-fed mice treated with CL, therefore suggesting that blunted BAT activation in WD-fed mice was not due to defective HNF4α or PPARα activity.

## Discussion

MASLD has become the most prevalent chronic liver disease worldwide, with a 30% prevalence in the adult population.^1^^,^^7^^,^^36^ Since diet-induced obesity is a major risk factors driving MASLD development, dietary-based preclinical models are used to investigate development of the human pathology. The so-called “western diets”, which are high in fat and carbohydrates, are generally accepted as relevant models.^14^^,^^16^ However, mounting evidence shows that mouse models housed at a suboptimal, cold-stress-inducing, ambient temperature (22°C) do not recapitulate human metabolic pathologies.^23^^,^^37^^,^^38^^,^^39^^,^^40^ Combining diet-induced obesity with thermoneutral housing has emerged as a promising method of refining the mouse model of MASLD. However, previous studies of C57Bl6 mice have induced MASLD using a high-fat diet combined with TN housing, and have failed to achieve robust hepatic fibrosis, a key feature of human MASLD.^26^^,^^30^ In the present study, we combined TN housing with a WD enriched in fat, sucrose, and cholesterol and observed consistent aggravation of diet-induced metabolic perturbations. WD-fed TN-housed mice presented with higher body weight and adiposity, and decreased glucose tolerance, compared to WD-fed RT-housed mice. Interestingly, WD-fed TN-housed mice had higher hepatic steatosis, compared to their RT-housed counterparts. These mice even developed liver inflammation and fibrosis—confirmed by both gene expression and histological studies—thereby confirming that our MASLD model mirrored pathological and molecular events consistent with human pathogenesis. Our results are consistent with those of Morrow et al.*,*^28^ which also demonstrated that TN housing significantly promoted histological and molecular MASLD features induced by a high-fat high-fructose diet. However, several other authors have reported no significant differences in steatosis or fibrosis development between WD-fed mice housed at TN or RT.^27^^,^^29^ This discrepancy might be due to differences in the duration of the dietary interventions, since our study was performed for a relatively short duration (13 weeks), in order to assess early MASLD-MASH transition, while other published studies cited above have been performed for longer periods (16–42 weeks). Thus, we suggest that the impact of TN on MASLD development might therefore be higher at the beginning of the pathology than at later stages. Another possible explanation could be that specific nutrient(s) within the different diets might interact(s) with the housing temperature to promote inflammation and fibrosis in the liver. Indeed, a recent study highlighted the critical role of diet composition in modeling the different steps of MASLD progression at thermoneutrality.^41^

Interestingly, our study revealed that TN housing decreased the intra-group variability in response to WD feeding, compared to housing at RT. This difference was observed for most investigated physiological parameters, including body weight, plasmatic enzymes, liver steatosis and inflammation, and hepatic gene expression. At the hepatic transcriptome level, unsupervised statistical analysis clustered 4 WD-fed RT-housed animals closer to the CD-fed mice than to the TN-housed WD-fed mice. Moreover, the genes with the most heterogeneous responses at RT were involved in inflammation and fibrosis development, consistent with phenotypical observations. Thus, in our experimental conditions, TN-housing induced a more homogenous response to the WD than RT-housing. We believe that this finding could have potential implications in optimizing murine models of MASLD as it suggests that TN-housing could allow significant reduction of the time of the dietary intervention and/or of the number of animals necessary to model MASLD by reducing the interindividual variability in response to dietary challenges. Our results however need to be confirmed using other housing conditions. The number of mice per cage indeed influences thermoregulation through regulation of *Ucp1* expression in the BAT^42^ and grouped housing is one of the key experimental conditions influencing MASH-fibrosis development in rodents.^16^

In addition to alterations of intrinsic liver metabolism, dysfunctional WAT strongly contributes to MASLD initiation and progression. WAT is the major site of lipid storage, and releases lipids into the circulation to fuel other organs (especially the liver) in case of energy need, thereby regulating systemic energy homeostasis.^43^ In obesity, WAT becomes dysfunctional, leading to increased basal lipolysis, dysregulated adipokine secretion, and release of pro-inflammatory molecules. In the liver, these mechanisms yield increased hepatic lipid content and inflammation, which further promote insulin resistance.^9^ In the present study, we used our TN-housed MASLD pre-clinical model to investigate this metabolic cross-talk between adipose tissues and the liver.

We employed adrenergic stimulation—a widely used approach to induce white adipose tissue lipolysis and investigate its systemic consequences, including insulin secretion,^44^ brown adipose tissue activation,^11^^,^^44^ and hepatic metabolic alterations^11^^,^^12^—to assess whether the development of MASLD, modeled through the combination of a WD and thermoneutral housing, modulates hepatic responsiveness to β3-adrenergic stimulation. Previous studies reported that activation of β3-adrenergic signaling induces major changes in hepatic gene expression.^11^^,^^12^ Cold-induced adrenergic stimulation and FFA release from WAT has been shown to increase hepatic HNF4α activation, thereby increasing expression of its target genes involved in acylcarnitine metabolism which leads to increased circulating acylcarnitine levels.^11^ In addition, we recently identified hepatocyte PPARα as a key player of the adipose-to-liver dialog that is required for hepatic gene expression, ketogenesis, and FGF21 production in response to WAT lipolysis.^12^ Here, gene expression analysis revealed a set of hepatic genes regulated upon acute lipolysis stimulation, mostly in CD-fed mice and less in WD-fed mice, suggesting that these genes were less sensitive to CL in WD-induced MASLD. However, our current results showed that ketogenesis and FGF21 production—two processes under the transcriptional control of PPARα in hepatocytes^45^^,^^46^^,^^47^^,^^48^—were similarly induced after β3-adrenergic receptor stimulation in CD-fed and WD-fed mice, indicating that these two hepatocyte PPARα-dependent hepatic functions remained functional in WD-induced MASLD. These findings suggest that the WAT lipolysis-dependent activity of hepatic PPARα is intact in MASLD. Similarly, known CL-dependent HNF4α hepatic target genes were not altered in our model of WD, thus suggesting that the WAT lipolysis-dependent activity of hepatic HNF4α is also intact in MASLD. Thus, our study extends previous findings and shows altered hepatic gene expression in response to adipose lipolysis, highlighting a disrupted white adipose tissue to liver cross-talk during MASLD.

Our results revealed that the hepatic response to selective β3-adrenergic receptor agonist was altered in our MASLD mouse model despite the lack of β3-adrenergic receptor expression in the liver. Interestingly, we observed that WD-fed mice did not exhibit impaired induction of adipose lipolysis by acute activation of β3-adrenergic signaling, indicating that altered hepatic gene expression was not due to defective lipolysis. Other potential mechanisms for altered transcriptional response to CL in the liver include adipokines and cytokines which are secreted from WAT and influence directly or indirectly liver metabolism in MASLD.^9^ WAT-secreted adiponectin binds to AdipoR1 and AdipoR2 receptors in the liver leading to enhanced FA oxidation and decreased lipogenesis.^49^ In addition, adiponectin signaling in the liver inhibits MCP1 expression leading to reduced macrophage infiltration and thus reduced hepatic inflammation.^50^ BAT-derived factors such as batokines and lipid mediators can target the liver^51^^,^^52^^,^^53^ and might also influence hepatic gene expression in response to lipolysis in WD-induced MASLD. Neuregulin-4 is a batokine secreted by cold-activated BAT that has been shown to act directly on hepatocytes to decrease lipogenesis and increase FA oxidation.^51^^,^^54^ In response to β3 adrenergic stimulation, BAT produces Maresin 2, a bioactive lipid, which attenuates hepatic inflammation.^52^ Whether WAT and/or BAT secretomes contribute to the altered hepatic gene expression in response to CL requires further investigations.

Finally, our results revealed that BAT activation in response to β3-adrenergic signaling stimulation was reduced in WD-induced MASLD, suggesting altered cross-talk between the liver and the BAT. This finding is consistent with a previous report that diet-induced obesity mice show reduced uptake of thermogenesis substrates (especially triglyceride-rich lipoproteins) by the BAT during β3-adrenergic receptor stimulation.^44^ To ensure efficient thermogenesis, brown adipocytes also use other energy-rich substrates, including glucose,^55^^,^^56^^,^^57^ FFAs released by white adipocytes,^58^^,^^59^ and acylcarnitines.^11^ In our present study, we did not analyze substrate uptake into BAT; however, we did not detect differences in the triglyceride, glucose, FFA, and acylcarnitine plasma levels in WD-fed mice compared to CD-fed mice.

In response to acute adipocyte lipolysis, the transcription factors PPARα and HNF4α not only control hepatic gene expression but also influence BAT activation.^11^^,^^12^ Our present findings indicated that WD feeding did not drastically influence PPARα-, nor HNF4α-dependent signaling, suggesting that reduced BAT activation in WD-induced MASLD does not depend on hepatocyte PPARα or HNF4α activity.

As already suggested, we assumed that β3-adrenergic signaling activation promotes insulin secretion from pancreatic β cells.^44^^,^^60^ This response to CL depends on adipose lipolysis and is required for BAT activation.^44^ Upon activation of β3-adrenergic signaling, we found that plasma insulin levels were not altered in WD-fed mice and were even higher compared to CD-fed mice. However, despite a high increase of plasma insulin levels, BAT activation was reduced in mice with WD-induced MASLD, suggesting defective BAT insulin signaling. Accordingly, mice with diet-induced obesity exhibit insulin resistance in BAT, and reduced BAT glucose uptake in response to β3-adrenergic signaling activation.^61^ Moreover, in a study using mice with an inducible brown adipocyte-specific insulin receptor deletion, Heine et al. showed that insulin signaling in BAT is essential for energy-rich substrate uptake and thermogenesis by BAT in response to β3-adrenergic stimulation.^44^ Thus, although we cannot exclude other hepatic or extra-hepatic mechanisms, we suggest that insulin resistance in BAT might contribute to the reduced BAT activation in WD-induced MASLD.

In conclusion, our present findings demonstrated that the combination of thermoneutral housing and WD feeding produces an accelerated mouse model of MASLD, which could facilitate the elucidation of the molecular mechanisms underlying the steatosis-to-steatohepatitis transition, as well as the testing of therapeutic approaches. We further used this model to show that MASLD is associated with an altered hepatic response to white adipose tissue lipolysis, including changes in hepatic gene expression, enhanced insulin levels, and reduced BAT activation in response to β3-adrenergic stimulation. Future studies employing pharmacological inhibition or genetic deletion of β3 adrenergic receptor (ADRB3) in adipose tissue to block lipid mobilization could be valuable to better understand the inter-organ signaling during MASLD progression.^62^^,^^63^ Altogether, these results reveal an altered inter-organ communication in MASLD and provide a useful resource for the modeling of MASLD and metabolic dialog with adipose tissues.

### Limitations of the study

A main limitation of our study is the exclusive use of male mice, despite the sexually dimorphic nature of MASLD. While we demonstrated that thermoneutral housing combined with WD feeding promoted MASLD in males and revealed altered hepatic and BAT responses to β3-adrenergic stimulation, the underlying mechanisms remain unresolved. Further studies evaluating WAT and BAT secretomes and their specific contribution to the hepatic and BAT responses would likely provide mechanistic insights.

## Resource availability

### Lead contact

Further information and requests for resources and reagents should be directed to and will be fulfilled by the lead contact, Nicolas Loiseau (nicolas.loiseau@inrae.fr).

### Materials availability

This study did not generate new reagents or Mouse lines for this study.

### Data and code availability

• Microarray data have been deposited at GEO and are publicly available as of the date of publication. Accession numbers are listed in the key resources table. All data reported in this paper will be shared by the lead contact upon request.
• This paper does not report original code.
• Any additional information required to reanalyze the data reported in this paper is available from the lead contact upon request.

## Acknowledgments

This work was supported by the French Foundation for Medical Research FRM (Equipe FRM EQU202303016327). This work was also supported by grant from the French National Research Agency (ANR) IMAGINE (ANR-20-CE14-0038) and the Hepatomics FEDER program of Région Occitanie. We thank Anexplo (Genotoul, Toulouse), We-Met facility (Inserm U1297, Toulouse) for their excellent work on plasma biochemistry and Lhorane Lobjois (Cell Imaging facility, INSERM, Toulouse) for the help with histology quantification of Sirius red and α-SMA.

## Author contributions

Conceptualization, N.L., A.F., and H.G.; formal analysis, C.M.P.M., A.P., V.A.-B., Y.L., J.B.-M., E.M., B.C., S.L., T.L., H.G., S.E.-S., A.F., and N.L.; investigation, C.M.P.M., A.P., V.A.-B., F.L., F.B.-Y.-E., C.N., M.H., C.R., J.B., V.M., J.W., J.B.-M., Y.L., E.R.-B., E.M., R.F.-F., C.D., B.C., S.L., D.L., T.L., L.G.-P., H.G., S.E.-S., A.F., and N.L.; writing—original draft preparation, C.M.P.M., S.E.-S., A.F., and N.L.; writing—review and editing, S.E.-S, N.L., A.F., and H.G.; visualization, C.M.P.M., A.P., S.E.-S., and N.L.; supervision, S.E.-S, N.L., and H.G.; funding acquisition, N.L., S.E.-S., and H.G.; all authors have read and agreed to the published version of the manuscript.

## Declaration of interests

The authors declare no competing interests.

## STAR★Methods

### Key resources table

**Table:** 

REAGENT or RESOURCE	SOURCE	IDENTIFIER
**Antibodies**		

Anti-α-SMA	Abclonal	RRID: AB_2861755

**Chemicals, peptides, and recombinant proteins**		

CL 316,243	Sigma-Aldrich	Cat# C5976
SYBR Green	Applied Biosystems	Cat# 4367659
TRIzol Reagent	Invitrogen	Cat# TR118

**Critical commercial assays**		

FGF21 ELISA kit	Sigma-Aldrich	Cat# EZRMFGF21-26K
Sureprint G3 Mouse G v2 microarrays	Agilent Technologies	N/A
Hot star Taq DNA Polymerase	Quiagen	Cat# 203605
High Capacity cDNA RT kit	ThermoFisher	Cat# 4368813

**Deposited data**		

Microarray data	This paper	GEO: GSE279895
Microarray data	This paper	GEO: GSE279897
Microarray data	This paper	GEO: GSE280075

**Experimental models: Organisms/strains**		

C57BL/6J (WT, male)	Charles River	N/A

**Oligonucleotides**		

Oligonucleotide sequences are listed in Table S5		

**Software and algorithms**		

LinRegPCR (v2021.2)	Ruijter et al.^64^	http://LinRegPCR.nl
GraphPad Prism (version 10.43.10)	GraphPad	https://www.graphpad.com
Metascape	Zhou et al.^65^	http://metascape.org
R (v4.1.3)	R Core Team^66^	https://www.R-project.org/
Rstudio(v2023.06.0)	RStudio, PBC	https://www.rstudio.com/
Bioconductor	Huber et al.^67^	https://bioconductor.org/
tidyverse (v1.3.2)	Wickham et al.^68^	https://cran.r-project.org/package=tidyverse
pheatmap (v1.0.12)	Raivo Kolde^69^	https://cran.r-project.org/package=pheatmap
Illustrator(v29.2.1)	Adobe	www.adobe.com/Illustrator
QuPath-0.5.1	QuPath-0.5.1.Ink	N/A
Cellpose	Stringer et al.^70^	https://www.cellpose.org
ImageJ	NIH	
limma(v3.60.4)	Ritchie et al.^71^	https://bioconductor.org/packages/release/bioc/html/limma.html
Scan Control A.8.5.1	Agilent	https://www.agilent.com/
Feature Extraction software v10.10.1.1	Agilent	https://www.agilent.com/
AriaMx (v1.8)	Agilent	https://www.agilent.com/

### Experimental model and study participant details

#### Animal

All experiments were carried out in accordance with the European Guidelines for the Care and Use of Animals for Research Purposes and approved by an independent ethics committee (CEEA-86 Toxcométhique) under the authorization number 17430–2018110611093660 v3. The animals were treated humanely with due consideration to the alleviation of distress and discomfort. A total of 64 C57BL/6J male mice (8-9-week-old) were purchased from Charles Rivers Laboratories (L’Arbresle, France). All mice were housed on a 12 h light (ZT0-ZT12)/12 h dark (ZT12-ZT24) cycle in a ventilated cabinet at the specific temperature, either a standard temperature (RT = 21°C–23°C) or a thermoneutral temperature (TN = 28°C–30°C) throughout the experiment. Mice were allowed two weeks of acclimatization with free access to water and food (standard rodent diet (safe A04 U8220G10R) from SAFE (Augy, France)). Then, the mice were randomly divided into eight groups of 8 mice each. Half of the mice were housed at TN and the other half at RT. In each housing condition, two groups (*n* = 16, 4 cages of 4 mice) were fed a chow diet (CD, A04, Safe Diets) and the other two groups (*n* = 16, 4 cages of 4 mice) were fed a Western diet (WD, TD.88137, Envigo) for 13 weeks. WD contains 42% calories from fat, 42.7% calories from carbohydrates (mainly sucrose) and 15.2% calories from proteins and 0.2% cholesterol; or a CD contains 8.4% calories from fat, 19.3% calories from carbohydrates and 72.4% calories from proteins. Body weight, food and water intake were measured weekly. Our previous study with the same WD demonstrated that 15-week feeding was indeed sufficient to induce hepatic inflammation and mild-fibrosis.^15^ In the present study, we hypothesized that TN-housing would accelerate/worsen the hepatic phenotype and thus decided to slightly shorten the diet duration to 13 weeks. On the day of sacrifice, mice were fasted at ZT0. At ZT10, half of the mice was gavaged with the β3-adrenergic receptor activator (CL 316,243, 3 mg/kg body weight; Sigma Aldrich) or vehicle (0.5% carboxymethylcellulose in sterilized water) and sacrificed at ZT16, 6 h after treatment. This experimental design is illustrated in Figure S1.

#### Human datasets

The human cohorts used in Figure S5 were previously published and as described.^16^ Briefly, the UCAM-VCU super-cohort consisted of 136 patients with clinical diagnostic of MASLD and histology scores divided into control (*n* = 4) and MASLD (subclustered against fibrosis (mild (F0), *n* = 52; moderate (F1-F2), *n* = 50; severe (F3-F4), *n* = 30)). The EPoS database encompasses 168 patients with MASLD subclustered against fibrosis (mild (F0), *n* = 47; moderate (F1-F2), *n* = 64; severe (F3-F4), *n* = 57). A description of the bioinformatics and biostatistical analyses used to process next-generation sequencing datasets can be found in Vacca et al*.*^16^

### Method details

#### Oral glucose tolerance test and plasma insulin measurement

All experiments were performed using conscious mice. After 10 weeks of feeding, six mice per group were randomly chosen, then fasted for 6h before receiving an oral glucose load (2 g/kg body weight). Blood glucose was measured at the tail vein using an Accu-Check Performa glucometer (Roche Diabetes Care France, Mylan, France) at 30 min before and 0, 15, 30, 60, 90 and 120 min after the glucose load. At 30 min before and 15 min after glucose injection, 20 μL blood from the tip of the tail vein was sampled for measurement of plasma insulin concentration using HTRF serum kits (Cisbio, Codolet, France). Briefly, 5 μL serum was incubated overnight at 4°C with the two corresponding monoclonal antibodies. When dyes are in close proximity, donor excitation via a light source triggers a fluorescence resonance energy transfer (FRET). The fluorescence energy transfer was measured using a Tecan Infinite 500 plate reader (Tecan, Lyon, France). The results were analyzed against a standard curve fit with the four-parameter logistic (4 PL) model, following the instructions for the kit (GraphPad Prism, USA).

#### Blood and tissue sampling

Prior to sacrifice, the submandibular vein was lanced and blood was collected into lithium heparin-coated tubes (BD Microtainer, BD, Dutscher, Brumath, France). Then, mice were killed by cervical dislocation. Plasma was isolated by centrifugation (1500 × g, 15 min, 4°C) and stored at −80°C until biochemical analysis. Tissue samples (liver, subcutaneous and perigonadic white adipose tissue (WAT), brown adipose tissue (BAT), caecum) were collected, weighed, dissected (when necessary) and prepared for histology analysis or snap-frozen in liquid nitrogen and stored at −80°C until further analyses.

#### Biochemical plasma analysis

Plasma samples were assayed for aspartate transaminase (AST) and alanine transaminase (ALT). All biochemical analyses were performed with a ABX Pentra 400 biochemical analyzer (Anexplo facility, Toulouse, France). Blood glucose levels were measured from mandibular vein using AccuCheck Performa glucometer strips (Roche Diagnostics), while β-hydroxybutyrate content was measured using Optium β-ketone test strips bearing Optium Xceed sensors (Abbott Laboratories, Abbott Park, IL, USA).

Free carnitine and acylcarnitines were measured from plasma (10 μL) spotted on filter membranes (Protein Saver 903 cards; Whatman), dried, and then treated as reported by Chace et al.^72^ Briefly, acylcarnitines were derivatized to their butyl esters and treated with reagents of the NeoGram MSMS-AAAC kit (PerkinElmer). Their analysis was carried out on a Waters 2795/Quattro Micro AP liquid chromatography-tandem mass spectrometer (Waters, Milford, MA).

#### Liver neutral lipid analysis

Hepatic lipids contents were extracted as previously described.^73^ Briefly, tissue samples were homogenized in Lysing Matrix D tubes with 1 mL methanol/5 mM EGTA (ethylene glycol-bis(β-aminoethyl ether)-N,N,N′,N′-tetraacetic acid) (2:1, v/v) in a FastPrep machine (MP Biochemicals). Lipids (corresponding to an equivalent of 2 mg of tissue) were extracted in chloroform/methanol/water (2.5:2.5:2, v/v/v), in the presence of the following internal standards: glyceryl trinonadecanoate, stigmasterol, and cholesteryl heptadecanoate (Sigma-Aldrich, Saint-Quentin-Fallavier, France). Total lipids were suspended in 160 μL ethyl acetate, and the triglycerides, free cholesterol, and cholesterol ester were analyzed with gas-chromatography on a Focus Thermo Electron system using a Zebron-1 Phenomenex fused-silica capillary column (5 m, 0.32 mm i.d., 0.50 μm film thickness; Phenomenex, England), as previously described.^74^ The oven temperature was programmed to increase from 200°C to 350°C at a rate of 5 °C/min, and the carrier gas was hydrogen (0.5 bar). The injector and the detector were set to 315°C and 345°C, respectively.

#### Histology

Paraformaldehyde-fixed, paraffin-embedded liver tissue was sliced into 3 μm sections and stained with hematoxylin and eosin (H&E) or into 5 μm sections and stained with Sirius red or used for immuno-histochemistry. The staining was visualized with a light microscopy equipped with a Leica DFC300 camera. All liver sections were analyzed blindly. Liver steatosis was evaluated according to Kleiner.^75^ Steatosis was measured depending on the percentage of hepatocytes containing fat, where Grade 0 = less of 5% of hepatocytes containing fat in any section; grade 1 = 5%–32% of hepatocytes; grade 2 = 33%–65% of hepatocytes; grade 3 = up to 65% of hepatocytes. The degree of inflammation was appreciated by counting the inflammatory foci into 10 distinct areas at 200X for each liver slice (grade 0 = no foci; grade 1 = less of 2 foci per 200X field; grade 2 = 2 to 4 foci per 200X field; grade 3 = up to 4 foci per 200X field). Sirius Red staining was used for evaluation of fibrosis in the liver. Briefly, paraffin-embedded liver blocks (5 μm) sections were incubated with picrosirius red (#ab150681, Abcam, France) solution for 1h and quickly washed with acidified water (0.5% acetic acid). Collagen was selectively visualized as bright orange-red birefringent fibers Immunohistochemical staining of anti α-smooth muscle actin (α-SMA) (ABclonal, A17910) was also performed. Then, the peroxidase activity was revealed with diaminobenzidine (kit SPlink HRP Rabbit DAB, GIB Labs) and sections were counterstained rapidly with hematoxylin. Sirius red and ‬‬‬‬‬‬‬‬‬‬‬‬‬‬‬‬‬‬‬‬‬‬‬‬‬‬‬‬‬‬‬‬‬‬‬‬‬‬‬‬‬‬‬‬‬‬‬‬‬‬‬‬‬‬‬‬‬‬‬‬‬‬‬‬‬α-SMA staining was visualized with a light microscope equipped with a Nikon Eclipse 90i at 10× magnification. Fibrosis objectively quantified as percent surface area occupied by Sirius red stained collagen or by ‬‬‬‬‬‬‬‬‬‬‬‬‬‬‬‬‬‬‬‬‬‬‬‬‬‬‬‬‬‬‬‬‬‬‬‬‬‬‬‬‬‬‬‬‬‬‬‬‬‬‬‬‬‬‬‬‬‬‬‬‬‬‬‬‬α-SMA staining by image analysis using Fiji. Lipid droplets detection, measurement and distribution visualization was performed using Python Jupyter Notebooks. Lipid droplets were detected using Cellpose with a model trained for lipid droplet segmentation,^70^ applied at different scales (6.8 and 20 μm). The resulting label images are then filtered with a size bandwidth adapted to each scale. Then these different scales are merged by adding them successively in increasing order. Labels were added if less than 10% of their area is occupied by other labels from smaller scales. On the resulting multi-scale image labels area are measured, then converted to an equivalent diameter. From these measurements, LD diameter distribution was estimated for each tissue with a weighted kernel density estimation using the seaborn library.^76^ The weight used was the area of each detection.‬ ‬‬‬‬‬‬‬‬‬‬‬‬‬‬‬‬‬‬‬‬‬‬‬‬‬‬‬‬‬‬‬‬‬‬‬‬‬‬‬‬‬‬‬‬‬‬‬‬‬‬‬‬‬‬‬‬‬‬‬‬‬‬‬‬‬‬‬‬‬‬‬‬‬‬‬‬‬‬‬‬‬‬‬‬‬‬‬‬‬‬‬‬‬‬‬‬‬‬‬‬‬‬‬‬‬‬‬‬‬‬‬‬‬‬‬‬‬‬‬‬‬‬‬‬‬‬‬‬‬‬‬‬‬‬‬‬‬‬‬‬‬‬‬‬‬‬‬‬‬‬‬‬‬‬‬‬‬‬‬

#### Gene expression

Total cellular RNA from liver and BAT was extracted with Trizol Reagent (Molecular Research Center, Inc., Cincinnati, OH, USA). RNAs were quantified using a nanophotometer (Implen). Total RNA samples (2 μg) were then reverse transcribed using the High-Capacity cDNA Reverse Transcription kit (Applied Biosystems) for real-time quantitative polymerase chain reaction (qPCR) analyses. The primers used for the SYBR Green assays are presented in Supplementary (Table S5). Amplifications were performed on a Stratagene Mx3005P thermocycler (Agilent Technology, Santa Clara, CA, USA). The qPCR data were normalized to the level of TATA-box binding protein (TBP) messenger RNA (mRNA), and analyzed with LinRegPCR software to determine mean efficiency (NO), which was calculated as follows: NO = threshold/(Eff meanCq), where Eff mean: mean PCR efficiency, and Cq: quantification cycle.

Microarray experiments were conducted on *n* = 7 mice per group (3 or 4 per cage). Gene expression profiles were performed at the GeT-TRiX facility (GénoToul, Génopole Toulouse Midi-Pyrénées, France) using Sureprint G3 Mouse GE v2 microarrays (8 × 60K, design 074809, Agilent Technologies) following the manufacturer’s instructions. For each sample, Cyanine-3 (Cy3) labeled cRNA was prepared from 200 ng of total RNA using the One-Color Quick Amp Labeling kit (Agilent technology) according to the manufacturer’s instructions. Then purification was performed by Agencourt RNAClean XP (Agencourt Bioscience Corporation, Beverly, Massachusetts, USA). Dye incorporation and cRNA yield were checked using Dropsense 96 UV/VIS droplet reader (Trinean, Gent, Belgium). A total of 600 ng of Cy3-labeled cRNA were hybridized on the microarray slides following the manufacturer’s instructions. Immediately after washing, the slides were scanned on Agilent G2505C Microarray Scanner using Agilent Scan Control A.8.5.1 software. The fluorescence signal was extracted using Agilent Feature Extraction software v10.10.1.1 with default parameters. All experimental details and microarray data are available in NCBI’s Gene Expression Omnibus^77^ and are accessible through Gene Expression Omnibus (GEO: GSE279895, GSE279897, GSE280075).

### Quantification and statistical analysis

All data are presented as means ± standard error of the mean (SEM). Statistical analysis on biochemical and qPCR data were performed using GraphPad Prism version 9 for Windows (GraphPad Software, San Diego, CA). One-way ANOVA was performed followed by appropriate post-hoc tests (Sidak’s multiple comparisons test) when differences were found to be significant (*p* < 0.05). For histological scores, non-parametric test (Kruskall-Wallis) were used. Significance were indicated by: ∗ or ^#^ for *p* < 0.05, ∗∗ or ^##^ for *p* < 0.01, ∗∗∗ or ^###^ for *p* < 0.001, ∗∗∗∗ or ^####^ for *p* < 0.0001.

Microarray data analyses were performed using R (R Core Team^78^) and Bioconductor packages,^67^ as described in GSE accession GEO: GSE279895, GSE279897, GSE280075. Raw data (median signal intensity) were filtered, log2 transformed and normalized using quantile method qsmooth method.^79^ A model was fitted using the limma ImFit function.^71^ Pairwise comparisons between biological conditions were applied using specific contrasts. A correction for multiple testing was applied using the Benjamini-Hochberg procedure (BH),^80^ to control the False Discovery Rate (FDR). Probes with FDR ≤0.05 were considered to be differentially expressed between conditions. Hierarchical clustering was applied to the samples and the differentially expressed probes using 1-Pearson correlation coefficient as distance and Ward’s criterion for agglomeration. The clustering results are illustrated as a heatmap of expression signals. The enrichment of Gene Ontology (GO) Biological Processes was evaluated using Metascape functions.^65^

## Supplemental information

Document S1. Figures S1–S7 and Tables S2 and S5

Table S1. List of genes and GO term enrichment analysis contained in each cluster of the microarray analysis of liver gene expression to identify biological processes sensitive to housing temperature under CD or WD feeding, related to Figure 2 Metabolic pathways (summary) were considered as significantly enriched when *q* value <0.01.

Table S3. List of genes and GO term enrichment analysis contained in each cluster of the microarray analysis, to identify biological processes that were sensitive to CL treatment under CD or WD feeding, related to Figure 4 Metabolic pathways (summary) were considered as significantly enriched when *q* value < 0.05.

Table S4. List of genes and GO term enrichment analysis contained in each cluster of the microarray analysis of BAT gene expression revealed biological processes that were sensitive to CL treatment under CD or WD feeding, related to Figure 5 Metabolic pathways (summary) were considered as significantly enriched when *q* value < 0.05.
