# Supplementary material for: Thermoneutral housing worsens MASLD and reveals defective brown adipose tissue response to β3-adrenergic stimulation
Source: iScience. 2025 Jul 26;28(9):113221. doi: 10.1016/j.isci.2025.113221 (PMC12396296; doi:10.1016/j.isci.2025.113221)
Supplement: Document S1. Figures S1–S7 and Tables S2 and S5 [file mmc1.pdf]

iScience, Volume *28*

Supplemental information

Thermoneutral housing worsens MASLD and reveals defective brown adipose tissue response to β3-adrenergic stimulation

Céline Marie Pauline Martin, Arnaud Polizzi, Valérie Alquier-Bacquié, Marine Huillet, Clémence Rives, Charlène J. G. Dauriat, Justine Bruse, Valentine Melin, Claire Naylies, Yannick Lippi, Frédéric Lasserre, JingHong Wan, Rémy Flores-Flores, Justine Bertrand-Michel, Florence Blas-Y-Estrada, Elodie Rousseau-Bacquié, Thierry Levade, Hervé Rémignon, Dominique Langin, Etienne Mouisel, Sophie Lotersztajn, Benoit Chassaing, Laurence Gamet-Payrastre, Hervé Guillou, Sandrine Ellero-Simatos, Anne Fougerat, and Nicolas Loiseau

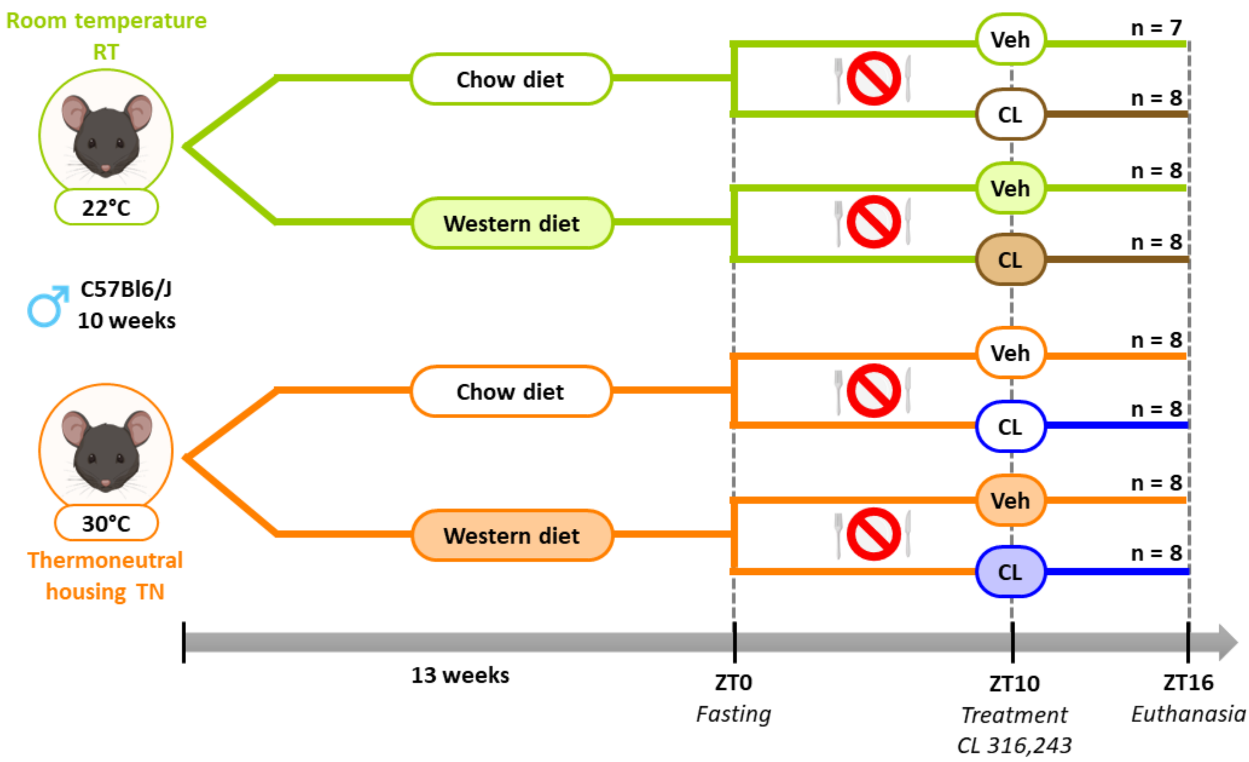

Supplementary Figure S1: Experimental design. WT male C57Bl/6J mice, aged 10 weeks, were housed at room temperature (RT; 22°C) or thermoneutral temperature (TN; 30°C) and fed a chow diet (CD) or western diet (WD) for 13 weeks (*n* = 7–8/group). On the day of sacrifice, mice were fasted at ZT0. At ZT10, half of the mice was gavaged with the β3-adrenergic receptor activator (CL 316,243, 3 mg/kg body weight) or vehicle (0.5% carboxymethylcellulose in sterilized water) and sacrificed at ZT16, six hours after treatment.

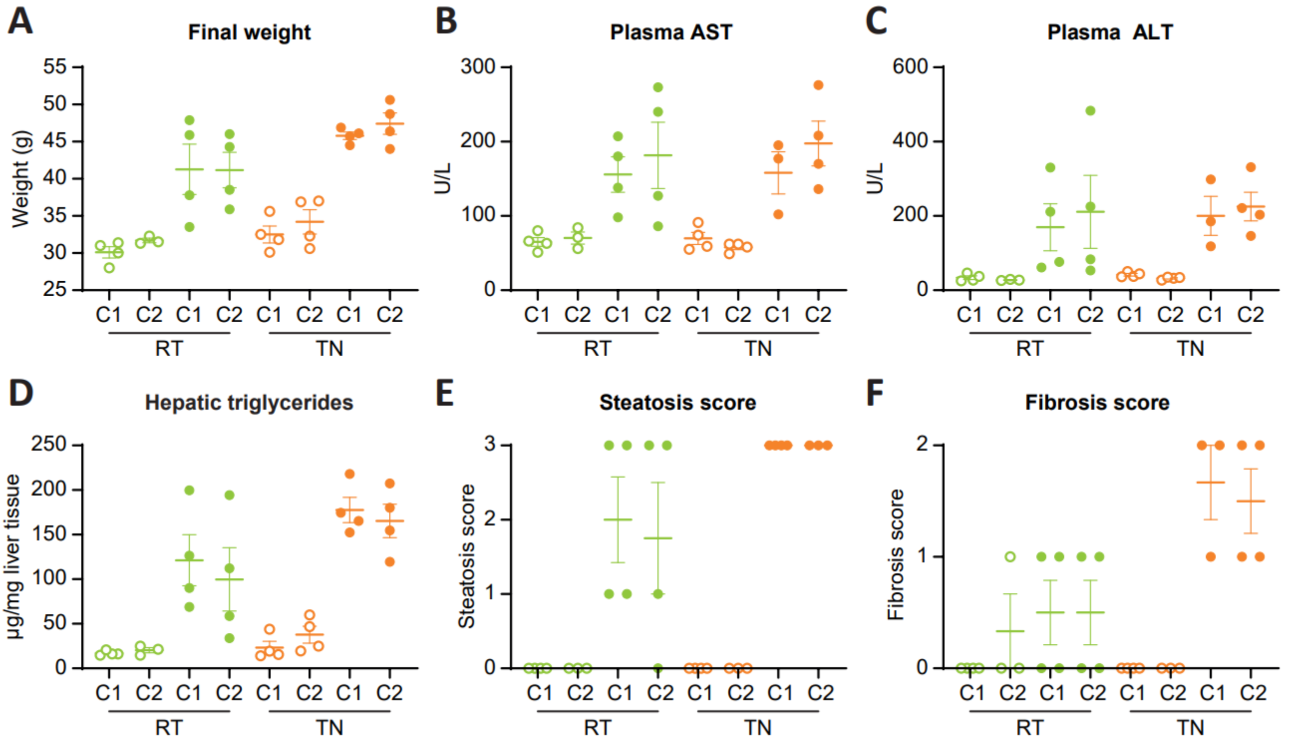

Supplementary Figure S2: Thermoneutral housing reduces the intra-group variability in WD-induced MASLD. (**A**) Final weight after 13 weeks of diet in each cage (C1 or C2) under room temperature (RT) or thermoneutral housing (TN). (**B, C**) Plasma ALT and AST activity. ALT alanine aminotransferase, AST aspartate aminotransferase. (**D**) Hepatic triglycerides extracted from the livers and analysed by gas-liquid chromatography. (**E**) Liver steatosis estimated on histological liver sections. Scoring: parenchymal involvement by steatosis <5%, 0; 5%-33%, 1; 33%-66%, 2; >66%, 3 (n=4/group). (**F**) Stellate hepatic cell activation estimated on histological liver sections. Scoring: lower than 3% of periportal area, 0; 3%-33%, 1; 34%-66%, 2; more than 66%, 3 (n=4/group).

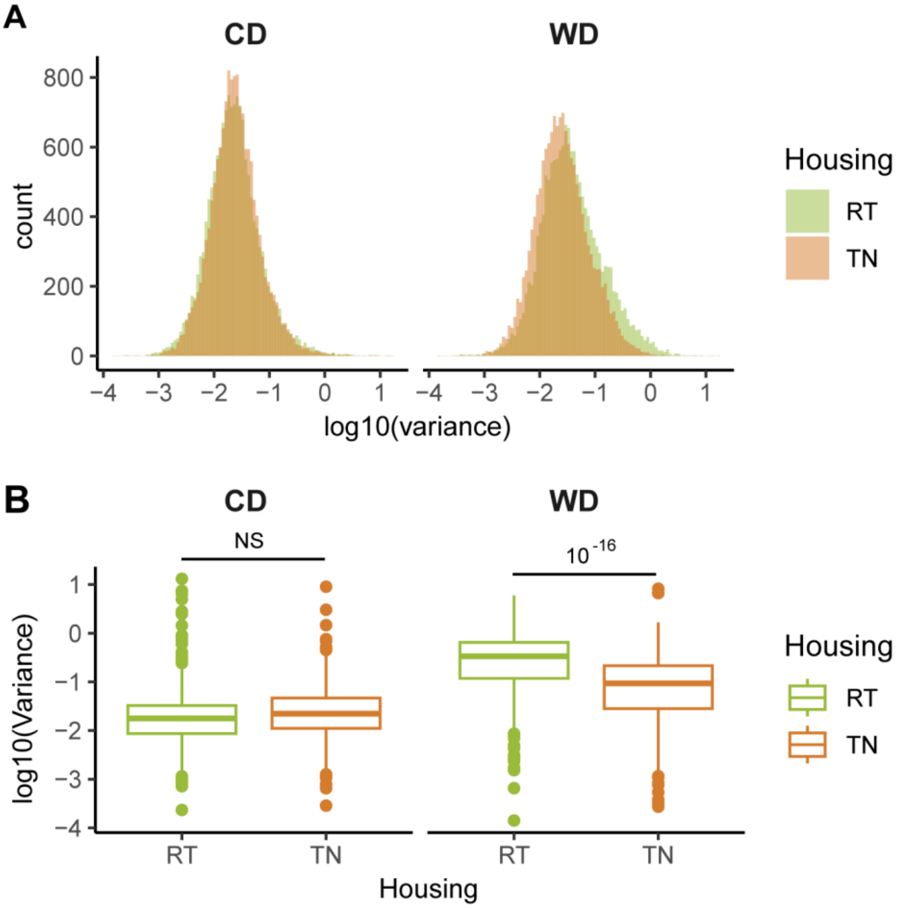

Supplementary Figure S3: Thermoneutral housing reduces the intra-group variability in WD-induced changes in hepatic gene expression. (**A**) Distribution of the variances per experimental group for all hepatic genes measured using microarrays. (**B**) Variances of the 449 non-homoscedastic hepatic genes.

A

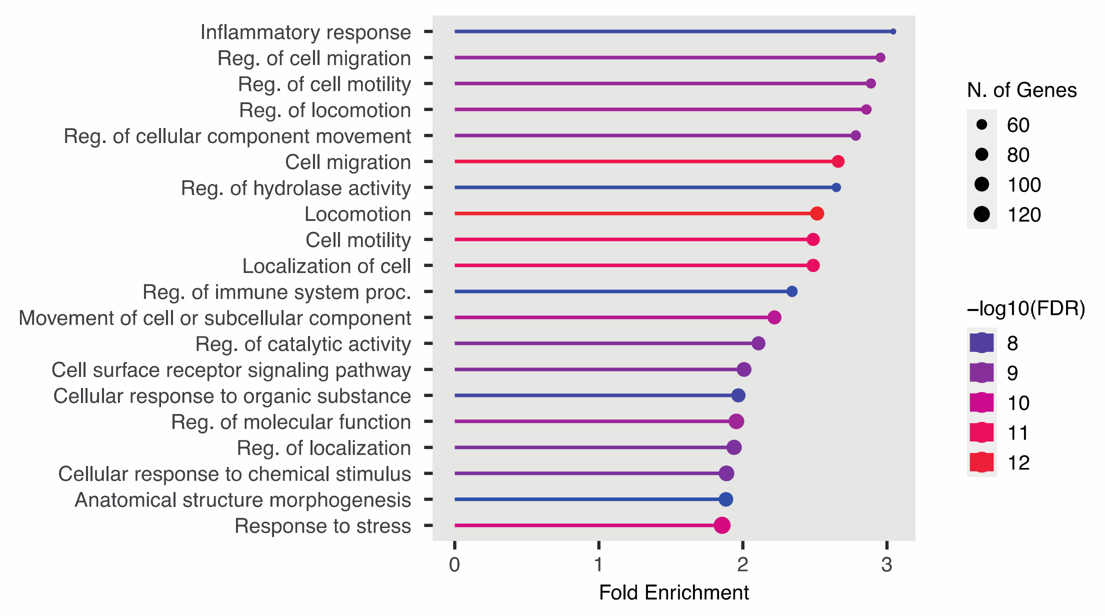

B

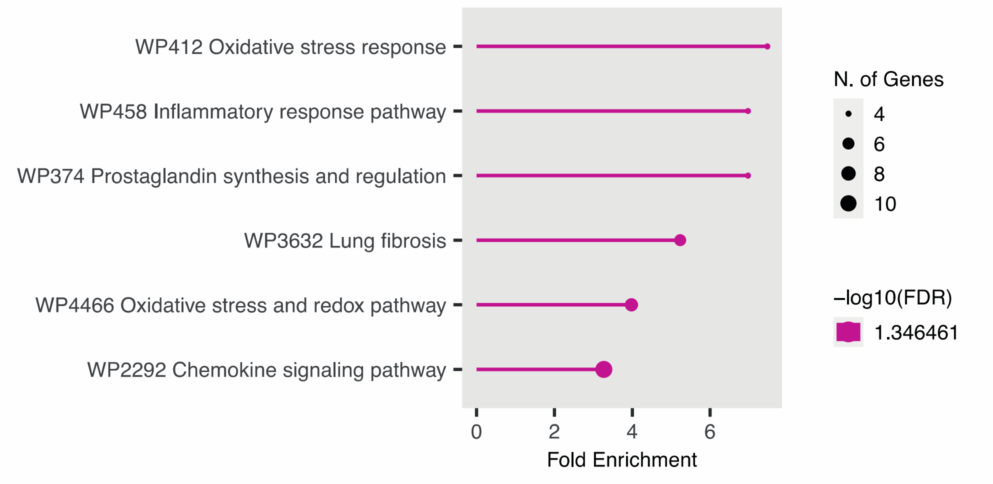

Supplementary Figure S4: Thermoneutral housing reduces the intra-group variability in WD-induced changes in hepatic gene expression. The most significantly enriched biological processes identified with the Metascape gene ontology algorithm are shown at the right of each profile. Briefly, hypergeometric tests were performed for each category in each cluster. (**A**) Distribution of the variances per experimental group for all hepatic genes measured using microarrays. (**B**) Variances of the 449 non-homoscedastic hepatic genes.

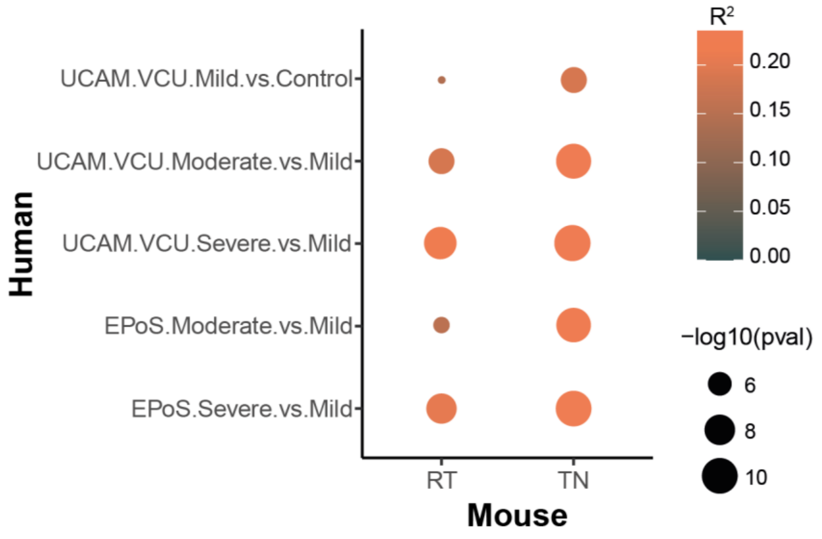

Supplementary Figure S5: Comparison of DEGs in mouse and human hepatic gene expression datasets. Log_2(fold-changes) of gene expression were correlated between mouse data (WD- vs. CD-fed mice at RT or TN) and human data (2 different human MASLD cohorts (UCAM.VCU and EPoS), comparing gene expression changes in liver samples from patients with different fibrosis stage, see methods).

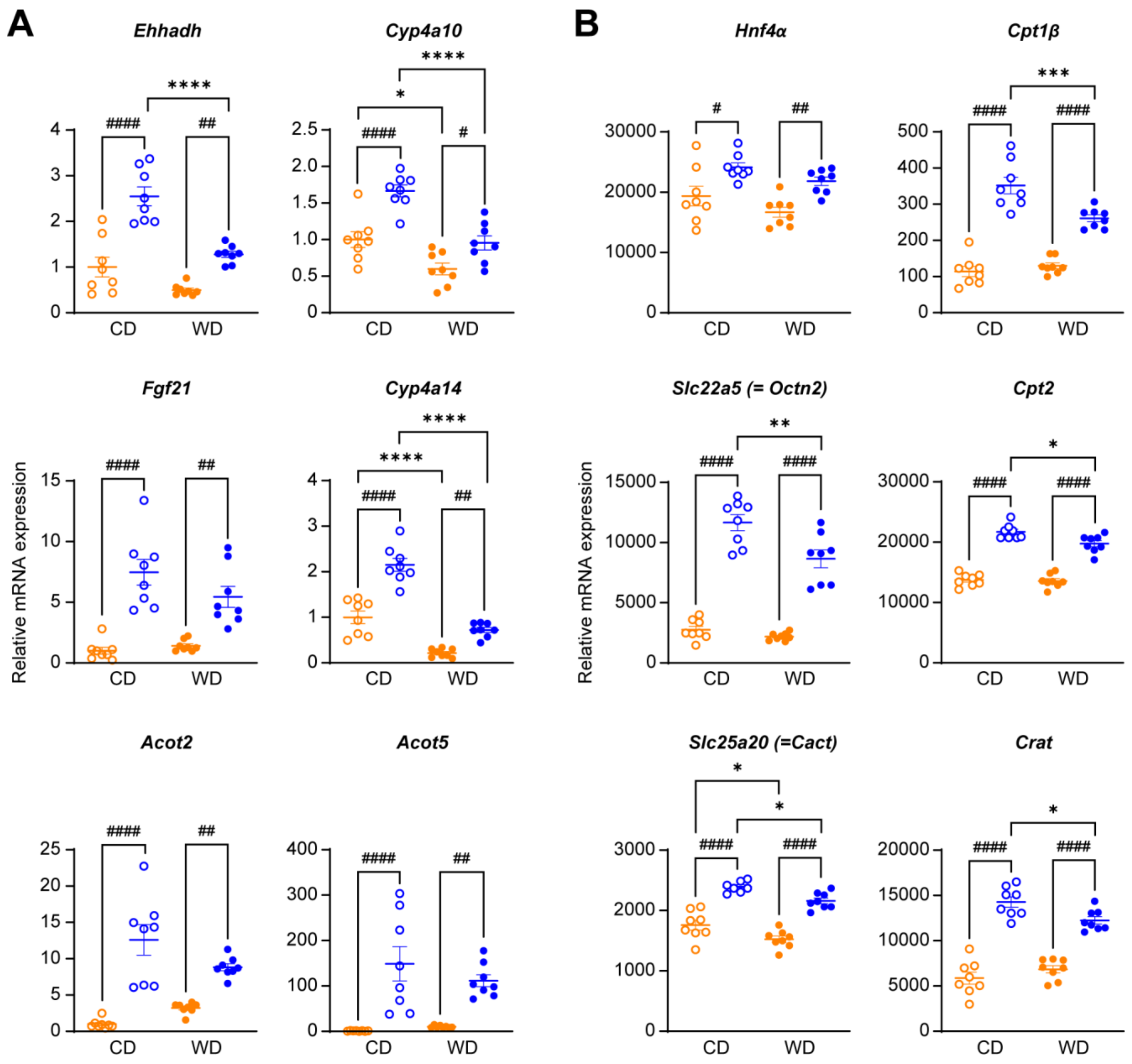

Supplementary Figure S6: β3-adrenergic stimulation increased hepatic PPARα and HNF4α target gene expression involved in β-oxidation and acylcarnitine metabolism respectively in CD- and WD-fed mice. (**A**) Hepatic mRNA expression of PPARα target genes (Acot2, Acot5, Cyp4a10, Cyp4a14, Ehhadh, Fgf21) measured by RT-qPCR. (B) Hepatic mRNA expression of HNF4α target genes (Hnf4α, Cpt1β, Octn2, Cpt2, Cact, Crat) measured by RT-qPCR.

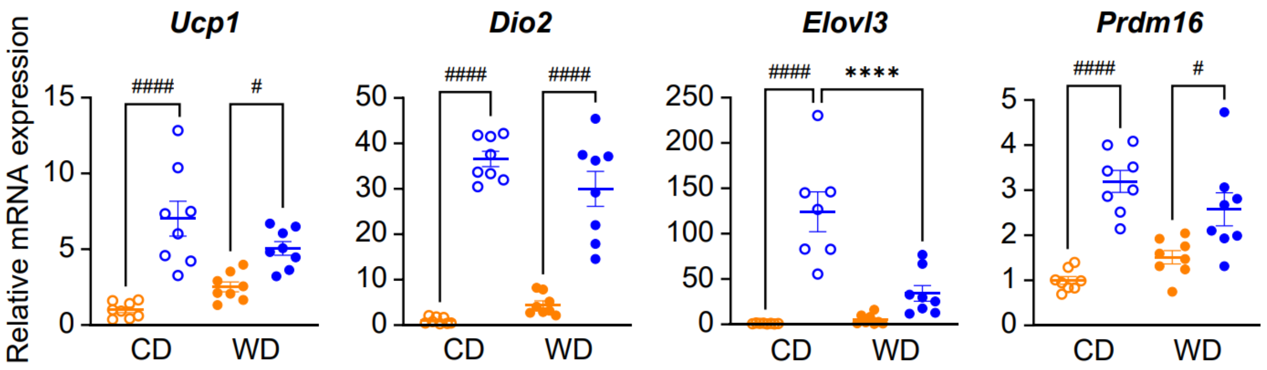

Supplementary Figure S7: β3-adrenergic signalling induced-BAT activation is blunted in MASLD condition. Related to Figure 5. mRNA relative expression of *Ucp1*, *Dio2*, *Elovl3* and *Prdm16* measured by qRT-PCR in the brown adipose tissue (BAT). Data are presented as the mean ± SEM for n = 8/group. * diet effect, # CL 316,243 effect. * or #P < 0,05, **P < 0,01, *** or ###P < 0,001, **** or ####P < 0,0001. Differential effects were analysed by analysis of variance (one-way ANOVA) with post hoc Šídák's test.

Supplementary Table S2: Descriptive statistics for selected metabolic parameters.

Parameter		CD RT	WD RT	CD TN	WD TN
Body weight (g)	Mean	30.8	41.2	33.3	46.6
	SD	1.4	5.4	2.7	2.1
	SEM	0.5	**1.9**	0.97	**0.7**
Plasma ALT (U/L)	Mean	31	190.3	36.9	214.6
	SD	7.7	154.4	7.1	77
	SEM	2.9	**54.6**	2.5	**29.1**
Hepatic triglycerides (µg/mg tissue)	Mean	18.3	110.5	30.5	171.5
	SD	3.9	60.9	17.2	31.5
	SEM	1.5	**21.5**	6.1	**11.1**

Supplementary Table S5: Oligonucleotide sequences for real-time qPCR.

Related to STAR methods

**Gene**	**NCBI RefSeq**	**Forward primer**	**Reverse primer**
Acot2	NM_134188	TGGAGTACTTTGAAGAAGCCGTG	CATTTTGACTTGGTTTCTCAGAAGG
Acot5	NM_145444	CATGGCTCTGGCTTATTATAAATATGAT	CCTTTGGAAATCCCTAGCAGG
Acta2	NM_007392	ACCCACCCAGAGTGGAGAA	GCTGTGCTGTCTTCCTCTTCA
Col1a1	NM_007742	CTCCGGCTCCTGCTCCTC	GGGTTTCCACGTCTCACCAT
Col3a1	NM_009930	TGGCACAGCAGTCCAACGTA	CATAGGACTGACCAAGGTGGCT
Cyp4a10	NM_010011	TCCAGCAGTTCCCATCACCT	TTGCTTCCCCAGAACCATCT
Cyp4a14	NM_007822	TCAGTCTATTTCTGGTGCTGTTC	GAGCTCCTTGTCCTTCAGATGGT
Dio2	NM_010050.4	AGCTTCCTCCTAGATGCCTACA	GGGAGCATCTTCACCCAGTTTA
Ehhadh	NM_023737	CGTCTCCTCGGTTGGTGTTC	ATTATCTTCTTTGCAGTATCTAGCTGCTT
Elovl3	NM_007703	GCCTCTCATCCTCTGGTCCT	TGCCATAAACTTCCACATCCT
Fgf21	NM_020013	AAAGCCTCTAGGTTTCTTTGCCA	CCTCAGGATCAAAGTGAGGCG
Il-1β	NM_008361.4	AAAAAAGCCTCGTGCTGTCG	GTCGTTGCTTGGTTCTCCTTGT
Mmp13	NM_008607	ACCTGGACAAGCAGTTCCAAAG	AGCTCATGGGCAGCAACAATAA
Prdm16	NM_027504.3	AAGGCGAGGGCGAGGAA	CATATTATTTACAACGTCACCGTCACT
Tnfα	NM_013693	TCCCCAAAGGGATGAGAAGTTC	GCGCTGGCTCAGCCACT
Ucp1	NM_009463.3	CCTGCCTCTCTCGGAAACAA	TGTAGGCTGCCCAATGAACA
